# Genome-Resolved Metagenomics and Metatranscriptomics Reveal Insights into the Ecology and Metabolism of Anaerobic Microbial Communities in PCB-Contaminated Sediments

**DOI:** 10.1021/acs.est.3c05439

**Published:** 2023-10-19

**Authors:** Hongyu Dang, Jessica M. Ewald, Timothy E. Mattes

**Affiliations:** Department of Civil and Environmental Engineering, 4105 Seamans Center, University of Iowa, Iowa City, Iowa 52242, United States

**Keywords:** polychlorinated biphenyls, Dehalococcoides, reductive dehalogenase, coexpression network

## Abstract

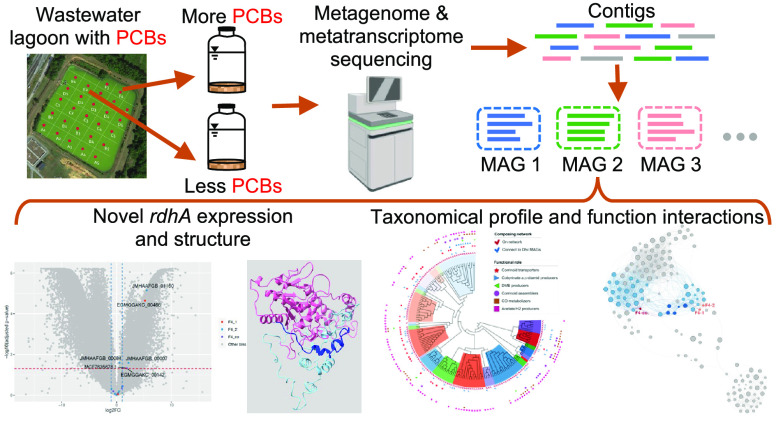

Growth of organohalide-respiring bacteria such as *Dehalococcoides mccartyi* on halogenated organics (e.g., polychlorinated biphenyls (PCBs)) at contaminated sites or in enrichment culture requires interaction and support from other microbial community members. To evaluate naturally occurring interactions between *Dehalococcoides* and key supporting microorganisms (e.g., production of H_2_, acetate, and corrinoids) in PCB-contaminated sediments, metagenomic and metatranscriptomic sequencing was conducted on DNA and RNA extracted from sediment microcosms, showing evidence of both *Dehalococcoides* growth and PCB dechlorination. Using a genome-resolved approach, 160 metagenome-assembled genomes (MAGs), including three *Dehalococcoides* MAGs, were recovered. A novel reductive dehalogenase gene, distantly related to the chlorophenol dehalogenase gene *cprA* (pairwise amino acid identity: 23.75%), was significantly expressed. Using MAG gene expression data, 112 MAGs were assigned functional roles (e.g., corrinoid producers, acetate/H_2_ producers, etc.). A network coexpression analysis of all 160 MAGs revealed correlations between 39 MAGs and the *Dehalococcoides* MAGs. The network analysis also showed that MAGs assigned with functional roles that support *Dehalococcoides* growth (e.g., corrinoid assembly, and production of intermediates required for corrinoid synthesis) displayed significant coexpression correlations with *Dehalococcoides* MAGs. This work demonstrates the power of genome-resolved metagenomic and metatranscriptomic analyses, which unify taxonomy and function, in investigating the ecology of dehalogenating microbial communities.

## Abstract 2

A genome-resolved metranscriptomics analysis revealed *Dehalococcoides* MAGs, expression of a novel reductive dehalogenase gene, and microbial interactions in a PCB-dechlorinating community.

## Introduction

Organohalide-respiring bacteria (OHRB) “breathe” halogenated organics to obtain energy for growth. As organohalides are often undesirable groundwater, sediments, or soil contaminants, bioremediation strategies (e.g., biostimulation, bioaugmentation, and natural attenuation) that take advantage of the unique OHRB lifestyle are popular and feasible for achieving contaminated site cleanup, especially for the chlorinated ethenes.^1−5^ However, OHRB-focused bioremediation strategies for polychlorinated biphenyls (PCBs) lag behind those for chlorinated ethenes.^6,7^

An OHRB genus of particular interest and relevance, *Dehalococcoides*, contains members that can grow with PCBs as an electron acceptor, hydrogen as an electron donor, and acetate as a carbon source.^7,8^ The key enzymes for organohalide respiration by *Dehalococcoides* are reductive dehalogenases (RDases) which contain a corrinoid cofactor involved in catalysis.^9^ RDases for PCB dechlorination identified from *Dehalococcoides* so far are bifunctional, catalyzing both PCB and tetrachloroethene (PCE) reduction, suggesting that precultivation with PCE would be a useful strategy for growing active biomass as PCB-dechlorinating *Dehalococcoides* are otherwise difficult to enrich.^10−14^ Because *Dehalococcoides* lead specialized lifestyles, their genomes are streamlined and lack many complete pathways to produce required substrates and cofactors (e.g., corrinoids) and metabolize harmful intermediates (e.g., carbon monoxide (CO)). These factors drive important interactions with microbial community members, as evidenced by syntrophic relationships found between *Dehalococcoides mccartyi* and hydrogen, acetate, and corrinoid producers and CO oxidizers.^15−18^ Sequencing technology advances have facilitated studies of interactions between *Dehalococcoides* and other microbes in PCB-contaminated marine sediments and enrichment cultures.^3,12,14,19,20^ Further deciphering the interactions between *Dehalococcoides* and indigenous microorganisms is important to improve and optimize halogenated pollutant bioremediation strategies, such as maintaining growth and reductive dechlorination activity of *Dehalococcoides* at contaminated sites.

In previous studies, we found 16S rRNA and reductive dehalogenase genes from *Dehalococcoides* in PCB-contaminated wastewater lagoon sediments showing dechlorinating activity without any historical report of PCE contamination.^21^ PCB dechlorination with concomitant growth of *Dehalococcoides* occurred in microcosms incubated under a natural attenuation scenario using sediments from the site.^22^ Nucleic acids (DNA and RNA) were extracted from a different set of less enriched site sediment microcosms with different PCB concentrations and subjected to metagenomic and metatranscriptomic sequencing.^23^

The purpose of this study was to recover additional metagenome-assembled genomes (MAGs), including *Dehalococcoides*, and perform a genome-resolved gene expression analysis of the sediment microbial community. We also aimed to assign functional roles to MAGs and perform genome-resolved network analysis to investigate relationships between *Dehalococcoides* and key members of the sediment microbial community. A genome-resolved metatranscriptomics approach could pave the way for future ecological analysis of dehalogenating microbial communities that reveals relationships between *Dehalococcoides* and the indigenous microbial community members and provides insights that could promote increased dehalogenation rates.

## Materials and Methods

### Microcosm Construction with PCB-Contaminated Sediment Samples

Four microcosms were prepared anaerobically in 160 mL serum bottles with a modified reduced anaerobic mineral medium (RAMM; 100 mL).^24,25^ The RAMM composition is described in Section S1. PCB-contaminated sediment samples (10 g each) from two different freshwater lagoon locations (F4 and E2) that previously showed dechlorination activity^22^ were added to duplicate bottles for each location (i.e., F4–1, F4–2, E2–1, and E2–2).

A mixture of organic acids (acetate, propionate, butyrate, and lactate) was added into the bottles with an initial concentration of 2.5 mM each.^25^ Site location, sediment sampling protocols, and microcosm preparation details exist elsewhere.^21,22,25^ Aroclor 1248 is the likely contaminating PCB mixture.^21^ No exogenous PCB congeners or microorganisms were added to the microcosms. The two sediment samples had different PCB concentrations. The high-PCB microcosms (HPCBM) contained an average of 28.04 ± 2.89 μg/mL PCBs, while the low-PCB microcosms (LPCBM) contained an average of 4.28 ± 1.05 μg/mL (*p* < 0.0001; Figure S1A).^23,26^

After 200 days of incubation, there were no significant changes in total PCB concentrations in either the HPCBM or the LPCBM (*p* > 0.05; Figure S1B). This is expected, as possible aerobic PCB hydroxylation activity was minimal in the anaerobic microcosms.

### PCB Extraction and Quantification

A liquid–liquid PCB extraction method was applied to microcosm slurry samples (2 mL), followed by addition of surrogate and internal standards as described previously.^22,25^ Each extracted batch of samples contained at least one laboratory blank comprised of hexane spiked with surrogate standards. PCBs in slurry extracts were quantified with a modified US EPA method 1668C.^27,28^ Surrogate standard recoveries and method blanks allowed us to evaluate extraction procedure efficiencies and quantify background PCB concentrations.^29^ The PCB congener data set is deposited in Iowa Research Online (DOI: 10.25820/data.006156).^26^ Statistical differences among PCB congener data were analyzed with an independent two-sided *t*-test with α = 0.05.

### DNA and RNA Extractions, High-Throughput Sequencing, and qPCR

DNA was extracted periodically during the experiment for the qPCR analysis of *Dehalococcoides* 16S rRNA gene abundance. DNA and RNA were extracted for high-throughput sequencing at day 200 of the experiment. Extraction and high-throughput sequencing of DNA and RNA from microcosms and qPCR analysis followed procedures described previously,^22,23^ with more detail in Section S1.

### Metagenome Assembly, Binning, Dereplication, Taxonomic Classification, and Annotation of MAGs

A previous metagenome coassembly approach, which combined reads from the four LPCBM and HPCBM metagenomic samples to assemble contigs, resulted in 62 MAGs^23^ that met the high-quality MIMAG standard (completion >90% and contamination <5%).^30^ Individual assembly approaches are also useful for recovering high-quality MAGs for genome-resolved analysis.^31^ Metagenomic reads trimmed with Trimmomatic (version 0.39)^32^ in each sample were also individually assembled into contigs sample by sample with Megahit (version 1.2.9; *k*-mer step 10 and maximum *k*-mer size 127).^33^

Short metagenomic reads in each sample were mapped to corresponding contigs with Bowtie2 (version 2.2.5) using default settings.^34^ Output SAM files were converted into sorted and indexed BAM files with samtools (version 1.6).^35^ Contigs longer than 1500 bases and the BAM file of each metagenomic sample were used for binning with Metabat2 (version 2.15) using default settings.^36^ Bin completeness and contamination/redundancy was determined with CheckM (version 1.2.2) and Anvi’o (version 7.1).^37,38^ This yielded 110 high-quality MAGs, including two *Dehalococcoides* MAGs only recovered from HPCBM (6_bin.5 from F4–1, 5_bin.153 from F4–2). Medium-quality MAGs (i.e., bins with completion ≥80% and contamination/redundancy >5%) were refined according to Anvi’o instructions (https://merenlab.org/2015/05/11/anvi-refine/), which yielded an extra 33 high-quality MAGs (Table S3). Additional 77 bins with completion ≥80% and contamination/redundancy ≤5% were designated as MAGs to build a reliable and nonredundant genome-resolved reference database that would minimize ambiguous hits during metagenomic and metatranscriptomic read mapping.

The 220 MAGs obtained from individual assembly along with the 62 coassembled MAGs were dereplicated to 158 nonredundant MAGs with dRep (version 3.4.2) using 95% and 99% average nucleotide identity (ANI) for primary and secondary clustering, respectively.^39^ The two dereplicated individually assembled MAGs classified as *D. mccartyi* were included in subsequent analysis along with the coassembled *D. mccartyi* MAG. Additional details about taxonomic classification, phylogeny, reads per kilobase per million mapped reads (RPKM) and covered fraction calculations, and MAG functional annotation are in Section S2.

A *Dehalococcoides* pangenomic analysis, including the *Dehalococcoides* MAGs recovered here, was conducted with the Anvi’o pangenome workflow^40^ as described in Section S2. Comparisons between reductive dehalogenase (RDase) genes in *Dehalococcoides* MAGs and genomes were performed and an RDase amino acid phylogenetic tree was constructed as described in Section S3.

### Metatranscriptomics Analysis and Coexpression Network

Nucleotide sequences from all MAG coding regions were concatenated and used to build an index with kallisto (version 0.46.1).^41^ Prokka-annotated^42^ rRNA and tRNA genes in MAGs were included. Trimmed metatranscriptomic reads were mapped to the index to quantify estimated counts and effective length of each gene using the quant mode of kallisto (version 0.46.1).^41^ Differential expression (DE) analysis between samples with high (treatment) and low (control) PCB concentrations was performed with the estimated counts using package limma (version 3.54.2)^43^ in R (version 4.2.2)^44^ as it performed well under different conditions.^45^

In previous work,^3,19,20,46−48^ co-occurrence networks were constructed using 16S amplicons to describe the relative abundance of microorganisms in the system (e.g., ASVs/OTUs). For metatranscriptomic data, the transcripts per million (TPM) value was used as an analogous representation for the overall gene expression of a MAG. TPM values were calculated for each of the 160 MAGs and used to build the coexpression network. To calculate the TPM of MAGs, estimated transcript counts and effective gene length mapped to each coding gene of an MAG were summed, while the mapping results for noncoding RNAs (i.e., rRNA and tRNA) were excluded. The TPM of a MAG was calculated as follows
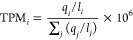
where *q*_*i*_ is the sum of estimated counts of each gene from an MAG, *l*_*i*_ is the sum of the effective length of each gene in an MAG. Estimated counts and effective length of each gene were kallisto outputs. ∑_*j*_ (*q*_*j*_/*l*_*j*_) corresponds to the sum of *q*_*i*_/*l*_*i*_ for each MAG.

Spearman’s rank correlation coefficients for TPM values of enriched genes (except rRNA and tRNA) in HPCBM from DE analysis and the TPM values of the MAGs were computed with the R package Hmisc (version 5.0–1).^49^ The *p* values were adjusted with the “BH” method. The coexpression network was constructed using the R package igraph (version 1.4.1).^50^ MAGs with correlation coefficients >0.8 and an adjusted *p*-value <0.05 to other MAGs were considered significantly correlated in the analysis. Functional roles were not considered when constructing the network. The network was then visualized in Gephi (version 0.10) with a force atlas layout.^51^

## Results and Discussion

### PCB Congener Profile Changes in Microcosms

**Figure 1:**
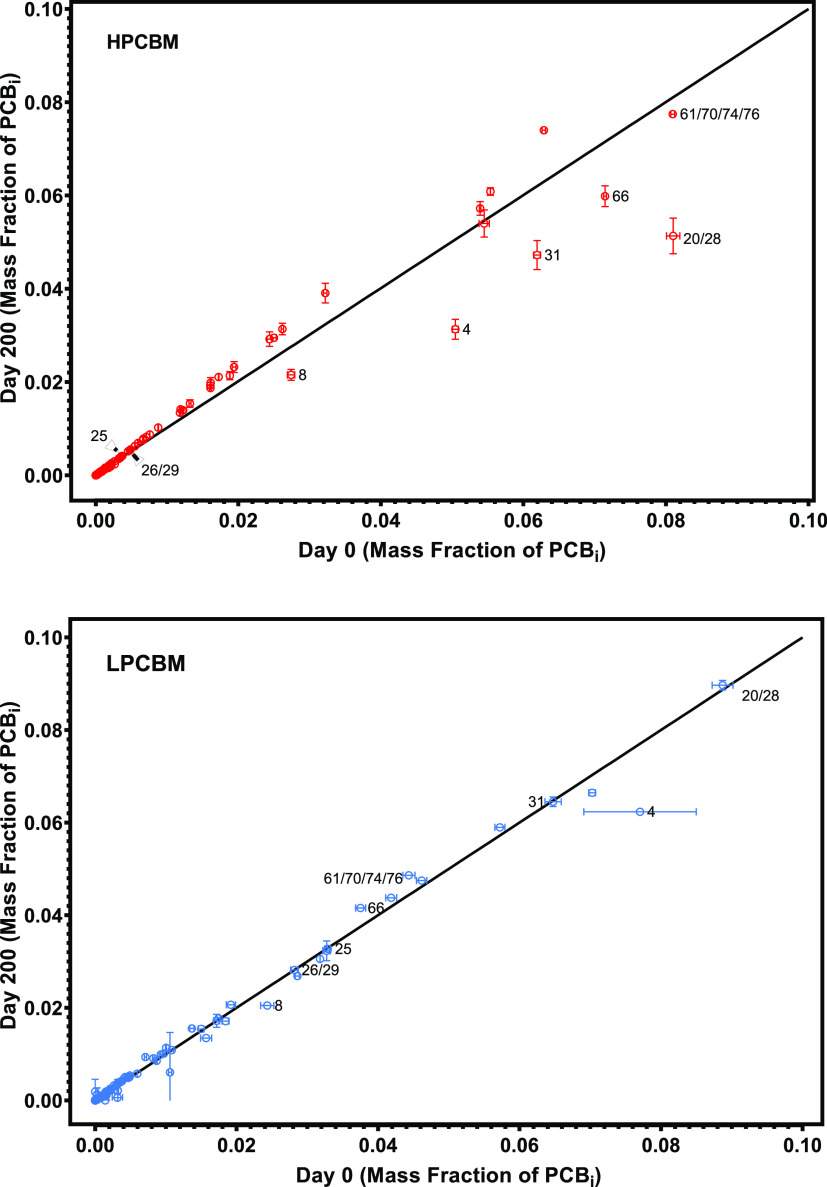
Profile comparisons of all 209 PCB congener mass fractions on days 0 and 200 in the HPCBM (top) and LPCBM (bottom). PCBi = PCB congener numbers from 1 to 209. The relationship between the PCB congener number and the IUPAC name can be found at www.epa.gov/sites/default/files/2015-09/documents/congenertable.pdf. When a PCB is labeled with multiple numbers (e.g., PCB 61/70/74/76), this indicates that these congeners coelute during gas chromatography separation of all 209 congeners. The black diagonal line on each plot shows where values would appear if the PCB congener mass fraction at day 0 equals the mass fraction at day 200. Congeners falling above or below the black line indicate a higher or lower mass fraction from day 0 to day 200 in the HPCBM or LPCBM, respectively. Data points represent the average PCB congener mass fraction measured in duplicate bottles. PCB congener number labels not shown in the plots were omitted for clarity. Error bars represent the variability in measurements collected from biological duplicates. The vertical error bar is the variability tested on day 200 and the horizontal error bar is the variability tested on day 0. The HPCBM data was adapted from ref (25). with the permission of Oxford University Press, copyright 2022.

Comparing PCB congener profiles at day 0 and at final sampling day 200 revealed greater differences in the HPCBM congener profile after 200 days^25^ than in the LPCBM, where few congeners diverged from the initial profile (Figure 1). In a previous microcosm study with these sediments over a 430-day incubation period, the mass fractions of tetrachlorinated PCB 66 and PCB 61/70/74/66 decreased, while mass fractions of trichlorinated dechlorination products PCB25 and PCB26/29 increased.^22^ Here, we observed significant decreases in PCB 66 (*p* = 0.0182) and PCB 61/70/74/66 (*p* = 0.0235) in the HPCBM but not in the LPCBM (Figures 1 and S2). Although mass fractions of expected dechlorination products PCB25 and PCB26/29 increased in the HPCBM but did not increase in the LPCBM (Figure S2), the changes were not statistically significant (*p* > 0.05). It is likely the shorter incubation time in this study compared to previous work (200 days vs 430 days)^22^ was insufficient to allow expected PCB dechlorination products to accumulate to statistically significant levels.

Mass fractions of trichlorinated PCB 31 and PCB 20/28 and dichlorinated PCB 8 also decreased after 200 days in the HPCBM compared to the LPCBM. Changes in mass fractions of these known PCB dechlorination products of higher chlorinated congeners, such as PCB 66,^52^ are further evidence of active PCB dechlorination in the HPCBM. PCB 4 is often considered a dechlorination end-product.^53^ Volatilization from the aqueous phase^54^ likely explains the greater abundance of PCB 4 at day 0 compared to day 200 in both the HPCBM and LPCBM (Figure 1).

#### *Dehalococcoides* Growth in Microcosms

We measured *Dehalococcoides* (*Dhc*) growth in the microcosms by quantifying the *Dhc* 16S rRNA gene abundance over time. Initially, little variability existed between *Dhc* 16S rRNA gene abundance in the HPCBM and LPCBM. The *Dhc* 16S rRNA gene abundance in all microcosms decreased from 2 × 10^4^ to 1.5 × 10^3^ copies/mL by day 21 (Figure S3). We observed a similar *Dhc* 16S rRNA gene abundance pattern with time in a previous microcosm study.^22^ The reason for the initial temporal decrease in *Dhc* 16S rRNA gene abundance is unknown but could possibly be explained by *Dhc* cells adapting to new incubation conditions in the microcosms.

*Dhc* growth in the HPCBM outpaced that of the LPCBM between days 83 and 211 (Figure S3). There was a significant increase in Dhc 16S rRNA gene abundance in the HPCBM between day 83 and day 211 (*p* = 0.014). The decrease in Dhc 16S rRNA gene abundance in the LPCBM between those time points was not significant (*p* = 0.119). On day 211, *Dhc* 16S rRNA gene abundance averaged 1.5 × 10^4^ gene copies/mL in HPCBM after 130 days of continuous increase. Conversely, the 16S rRNA gene abundance in the LPCBM averaged 1.2 × 10^3^ copies/mL at day 211. The mean *Dhc* 16S rRNA gene abundance significantly differed between the HPCBM and LPCBM at days 180 (*p* = 0.008) and 211 (*p* = 0.0003). DNA and RNA were extracted for sequencing at day 200, after *Dhc* 16S rRNA gene abundance in the HPCBM had significantly increased relative to that in the LPCBM.

In a previous microcosm study with the same sediments, the *Dhc* 16S rRNA gene abundance was 3–4 orders of magnitude greater^22^ (after 430 days incubation) than in this study (after 200 days incubation). Thus, we would expect additional *Dhc* growth in the HPCBM had we provided an additional incubation time.

### Recovery and Diversity of MAGs from Sediment Metagenomes

**Figure 2:**
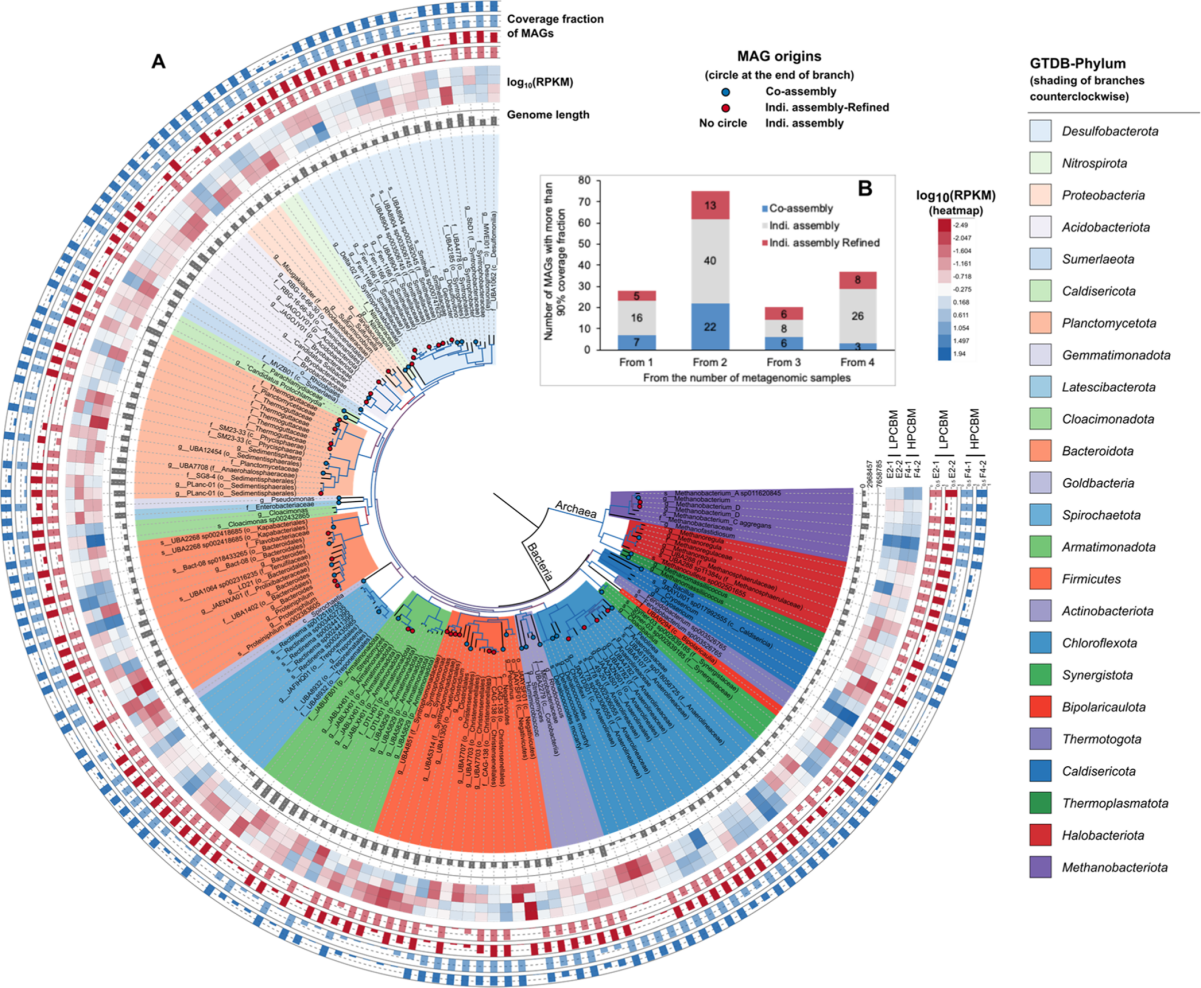
(A) Maximum likelihood phylogenetic tree of MAGs, taxonomy, genome length, log_10_(RPKM) and coverage fraction in metagenomic data. Blue circles at the end of tree branches depict coassembled MAGs, no circles depict individually assembled MAGs, and red circles depict MAGs with a refined individual assembly. The shading of the branches indicates the phylum. The prefix s_, g_, f_, o_, c_, and p_ in the taxa name represents the taxonomy rank at species, genus, family, order, class, and phylum levels. The innermost bar chart depicts the overall contig length in each MAG (gray bars). The heatmap shows the log_10_(RPKM) of MAGs in the HPCBM and LPCBM metagenomes. The bar charts in the outer circle show MAG coverage fractions in duplicate samples with a high (blue; HPCBM) or low (red; LPCBM) PCB concentration. (B) Distribution MAGs with more than 90% coverage fraction by the number of samples.

A combined individual and coassembly approach has previously been suggested for effective recovery of MAGs.^31^ The maximum MAG coverage fraction across metagenome samples ranges from 90 to 100%, with 132/160 MAGs covered by at least two metagenomic samples (out bar of Figure 2A,2B). Individual assembly contributed 90/160 dereplicated MAGs, followed by coassembly (38/160) and refined medium-quality MAGs (32/160). The analysis suggests that combining coassembly, individual assembly, and refining medium-quality bins was effective for recovering high-quality MAGs from metagenomes.

The 160 MAGs were classified into 24 phyla according to the Genome Taxonomy Database (GTDB)^55^ (Figure 2). The most abundant phyla in both the HPCBM and LPCBM were *Caldisericota*, *Chloroflexota*, *Methanobacteriota*, *Bacteroidota*, *Desulfobacterota*, *Spirochaetota*, and *Firmicutes*. Two other phyla, *Halobacteriota* and *Cloacimonadota* were only abundant in LPCBM. Phylum-level classifications of the MAGs were compared with those from a previous read-based taxonomic analysis,^23^ with the GTDB phylum shown as their corresponding NCBI phylum (Figure S4). There were 46 MAGs classified at a taxonomic level higher than that of the genus and 49 MAGs with no corresponding NCBI genus classification (Table S4), which suggests that they are potentially novel taxa. Many of the abundant MAGs have not been previously reported in PCB-degrading microbial communities harboring *Dehalococcoides*. Additional taxonomic analysis is provided in Section S4.

The genome-resolved approach used here contrasts with those of many previous metagenomic studies of organohalide-respiring microbial communities. Using read-based methods^2,56−58^ makes it challenging to establish clear links between phylogeny and function. Other studies that utilized metagenome assembly focused on *Dehalococcoides* MAG/genome recovery and RDase gene identification rather than recovering MAGs from other microbial community members.^10,12,19,59−61^ The few previous genome-resolved studies of organohalide-respiring microbial communities yielded between 7 and 37 MAGs.^62−64^ Here, our genome-resolved analysis yielded 160 MAGs in an effort to represent the sediment microbial community. This approach provides new insights about relationships between MAGs and guidelines on MAG recovery from organohalide-respiring microbial communities.

Although many high-quality MAGs were recovered from organohalide-respiring microbial communities in this study by analysis of short-read sequences (i.e., Illumina platform), reconstructing contigs and MAGs from short-read sequences is complicated, time-consuming,^65,66^ and potentially unreliable because of read mapping uncertainties.^67^ More recently developed long-read sequencing platforms can now routinely generate reads >10 Kb.^65−67^ Long reads are more suitable for de novo assembly and provide more complete MAGs or draft genomes for microbial identification.^65,67^ Long-read sequencing should be considered more frequently for future research involving recovery of MAGs from microbial communities.

#### *Dehalococcoides* Pangenomic Analysis

We compared the individually assembled *Dehalococcoides* MAGs from this study with 46 of 51 *Dehalococcoides* genomes and assemblies found on NCBI in April 2023 (including the coassembled *Dehalococcoides* MAG^25^) (Figure S5). *Dehalococcoides* MAG sizes ranged from 1.2 to 1.4 Mb, which are similar to complete *Dehalococcoides* genome sizes in NCBI (Figure S5). These MAGs contained between 1190 and 1448 coding genes, which suggests that these *Dehalococcoides* have streamlined genomes.^58,68^

Hierarchical clustering of single-copy genes (SCGs) in genomes/assemblies revealed the three distinct clades known as Pinellas, Victoria, and Cornell,^69^ which was also supported by the ANI between genomes/assemblies. The *Dehalococcoides* MAGs were placed in the Cornell subgroup (Figure S5). The ANI among the three *Dehalococcoides* MAGs was >99.8% and was >98% with PCB-dechlorinating strain CG4. A partial 16S rRNA gene was recovered from the coassembled *Dehalococcoides* MAG^25^ but not from the individually assembled *Dehalococcoides* MAGs.

A total of 27 RDase genes with >90% identity to each other were annotated in the *Dehalococcoides* MAGs (Figure S6). The individually assembled *Dehalococcoides* MAGs do not harbor any previously identified PCB dehalogenase genes (i.e., *pcbA1*, *pcbA4*, *pcbA5*,^10^ and *mbrA*(13)) and as previously reported for the coassembled *Dehalococcoides* MAG.^25^ Compared to a previous pangenomic analysis which compared the genomic and evolutionary characteristics of *Dehalococcoidia*,^70^ here, the pangenomic analysis focuses on previously identified PCB dehalogenase genes in other *Dehalococcoides* genomes/assemblies (further described in Section S5 and Table S5).

### Gene Expression in *Dehalococcoides* MAGs and Identification of a Novel, Expressed Reductive Dehalogenase Gene

**Figure 3:**
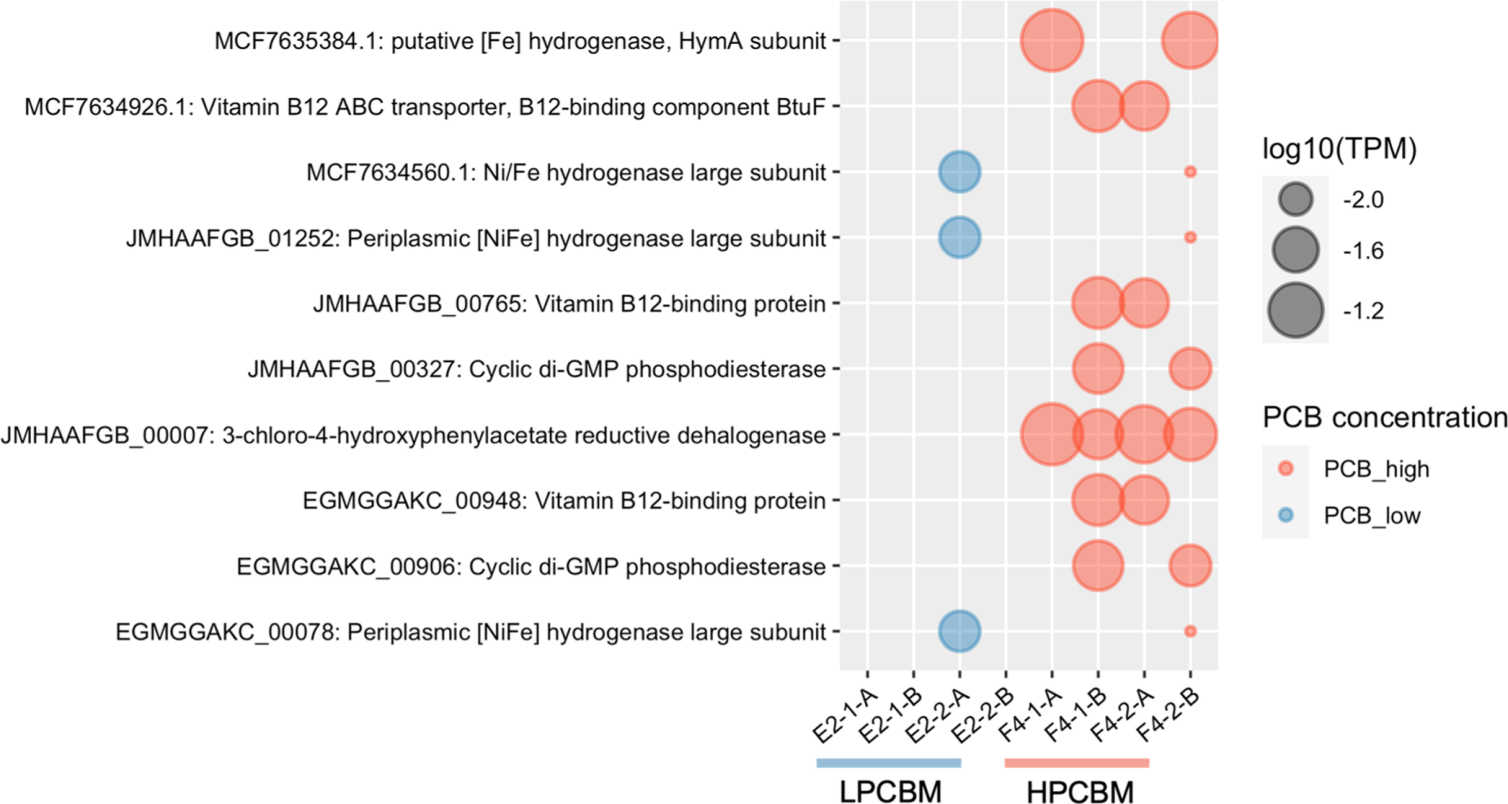
Log_10_ of transcripts per million (log_10_TPM) values for genes from *Dehalococcoides* MAGs expressed in at least two metatranscriptomics samples with high (red) or low (blue) PCB concentration.

We examined gene expression in the *Dehalococcoides* MAGs in metatranscriptomic samples (Figure 3 and Table S6). Two of the 27 annotated RDase genes (JHMAAFGB_00007 and JHMAAFGB_00005) were expressed in the HPCBM. JHMAAFGB_00007 was expressed in both HPCBM bottles (Figure 3), but JHMAAFGB_00005 was only expressed in one HPCBM bottle (Table S6). JHMAAFGB_00007 was uniquely present in one *Dehalococcoides* MAG (5_bin.153; labeled with *rdhA* in Figure S5). JHMAAFGB_00007 was not present in any other *Dehalococcoides* genomes/assemblies on NCBI, although it is in a variable region of the *Dehalococcoides* pangenome (Figure S5). Conserved RDase domains, including Pfam13486 (Fe–S binding domain) and TIGR02486 (corrin and Fe–S cluster-containing domain), were identified in these expressed RDase genes (Figure S7).

Genes encoding hydrogenases (e.g., MCF7635384.1, MCF7634560.1, JMHAAFGB_01252, and EGMGGAKC_00078) and vitamin B12 transporting/binding proteins (e.g., MCF7634926.1, JMHAAFGB_00765, and EGMGGAKC_00948) were also expressed in the HPCBM (Figure 3). A differential expression analysis revealed that the RDase gene (JHMAAFGB_00007) and a corrinoid salvaging gene (*cobV*/*cobS*; AGIKBDMD_00194) were significantly expressed in the HPCBM compared to the LPCBM (Figure S8). These expressed genes imply dehalogenation activity in the HPCBM.

In other PCB dehalogenation studies using transcriptomics, the *Dehalococcoides* cell density was several orders of magnitude higher than that in our study. Thus, a wide range of RDase gene expression from *Dehalococcoides* genomes was detected, but only those that were significantly expressed were identified as PCB dehalogenase genes.^10,12,13,71^ Here, RNA was extracted from microcosms that were far less enriched for *Dehalococcoides*. It is possible that other RDase genes were expressed in HPCBM at levels that were undetectable by metatranscriptomic sequencing despite the robust sequencing coverage (45–71 million sequences per sample).

Temporal analysis of RDase gene expression in some *Dehalococcoides* strains (e.g., CBDB1) show a peak in expression during early growth stages that declines in later stages,^72−75^ while in other strains, RDase gene expression remains at constant levels until the organohalide substrate is depleted.^76^ Here, we evaluated RDase gene expression at one time point in the HPCBM, likely in the early growth phase, according to qPCR data. A time-series metatranscriptomics analysis could have revealed JHMAAFGB_00007 and JHMAAFGB_00005 expression dynamics during growth phases and whether these were the only RDases significantly expressed temporally, as seen in other PCB-dechlorinating *Dehalococcoides* strains.^76^

A phylogenetic tree of RDase genes from *Dehalococcoides* MAGs and selected genes from the Reductive Dehalogenase Database^69^ (RDD) revealed that RDase genes in *Dehalococcoides* MAGs grouped within 26 different ortholog groups (OGs) (Figure S9). Two OGs (OG14 and OG15) contained RDase genes retrieved from *Dehalococcoides* MAGs (JHMAAFGB_00380 and JHMAAFGB_00392, respectively), and RDase genes cloned from the same site (Figure S9).^21,22^ The RDase genes (JHMAAFGB_00380 and JHMAAFGB_00392) were >90% identical to RDase genes retrieved from the clone library (Figure S6).

The expressed RDase gene (JHMAAFGB_00007) was in a small cluster away from most RDase genes in the RDD. An RDase gene from 1,2,3,4-tetrachlorodibenzo-p-dioxin-dechlorinating *Dehalococcoides* strain H1–3–2.001^61^ (PKH47860.1, OG342) grouped nearest to JHMAAFGB_00007 (Figure S9). BLAST analysis indicated that the pairwise amino acid identity between JHMAAFGB_00007 and PKH47860.1 was 48.18%. JHMAAFGB_00007 was annotated as 3-chloro-4-hydroxyphenylacetate reductive dehalogenase (*cprA*) with the Prokka default database, although the pairwise amino acid identity was 23.75%. The *cprA* gene product from *Desulfitobacterium hafniense* transforms 3-chloro-4-hydroxyphenylacetate to 4-hydroxyphenylacetate and dehalogenates chlorinated phenols.^77,78^

**Figure 4:**
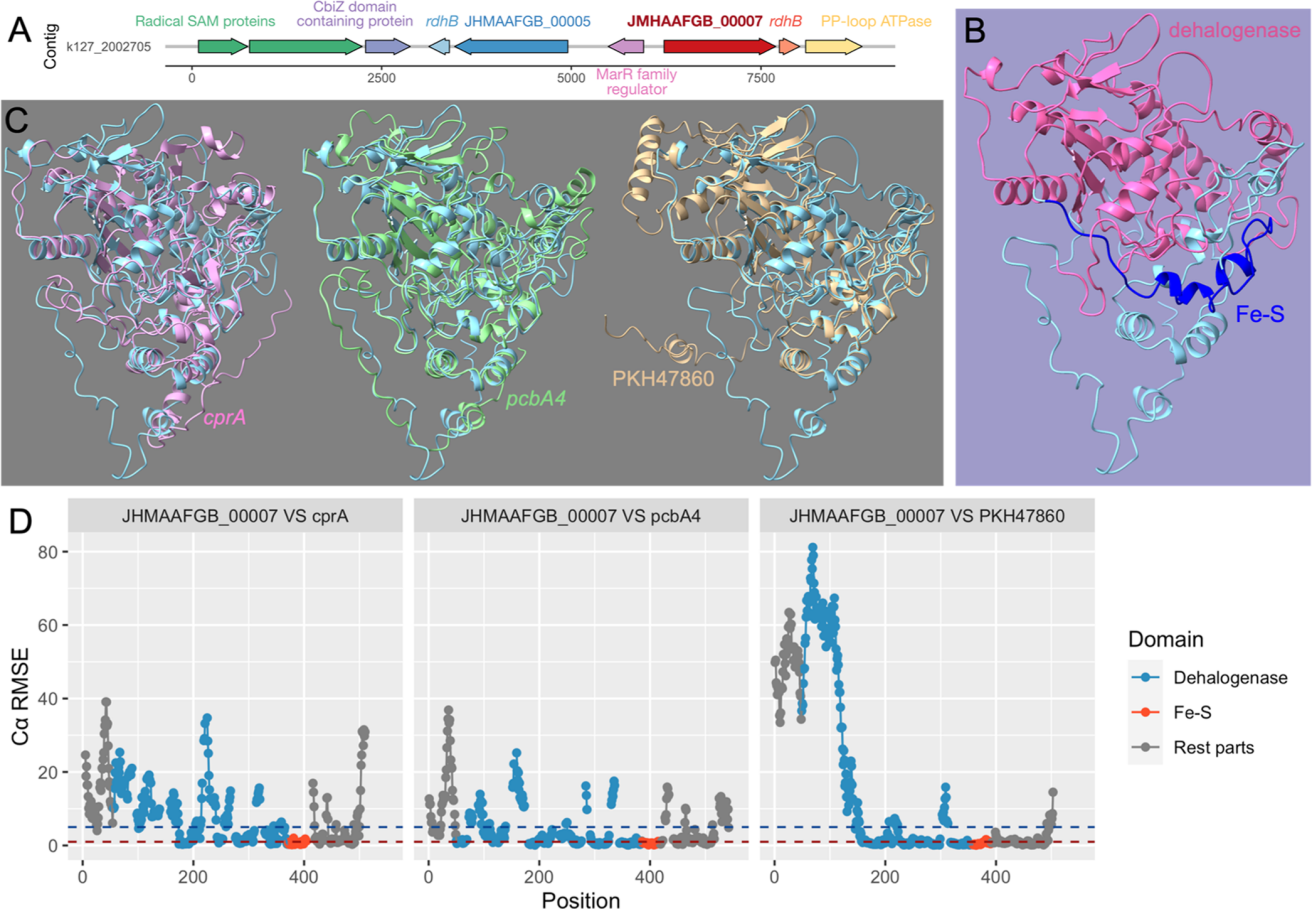
(A) Genes encoded on the contig containing the expressed *rdhA* (JHMAAFGB_00007). (B) The predicted tertiary structure of the JHMAAFGB_00007 product with the dehalogenase domain is highlighted in pink and the Fe–S domain in dark blue. (C from left to right) Comparison of the predicted JHMAAFGB_00007 product structure to *cprA*, *pcbA4*, and PKH47860 (D) The root-mean-square error at each atomic position (Cα RMSE) is between the expressed *rdhA* (JHMAAFGB_00007) and the other three predicted structures with highlighted dehalogenase (blue) and Fe–S (orange) domains. Discontinuities represent missing positions for structure matching. The blue dashed line represents Cα RMSE = 5, and the red dashed line represents Cα RMSE = 1.

Analysis of genes on the contig that harbored the significantly expressed *rdhA* (JHMAAFGB_00007) revealed that the second expressed *rdhA* (JHMAAFGB_00005) was encoded nearby (Figure 4A). Genes that encode dehalogenase anchoring protein (*rdhB)* were also located as expected near JHMAAFGB_00007 and JHMAAFGB_00005. None of the genes on this contig had annotated functions related to horizontal gene transfer. The predicted tertiary structure of the JHMAAFGB_00007 product (Figure 4B) was compared with the predicted tertiary structures of *cprA*, *pcbA4*, and PKH47860.1 products (Figure 4C,D). The predicted dehalogenase domain of the JHMAAFGB_00007 product was distinct from the other predicted protein structures. However, between positions 157 and 289 (within the dehalogenase domain), the predicted structures of the JHMAAFGB_00007 and PKH47860.1 products were similar, as shown by the small root-mean-square error at each atomic position values (Cα RMSE). This suggests that the JHMAAFGB_00007 product catalyzes the removal of chlorine from chlorinated aromatics (e.g., PCBs). Consequently, the lines of evidence presented thus far suggest that the significantly expressed RDase gene (JHMAAFGB_00007) is novel and has a high potential to encode a PCB dehalogenase. However, further experimental evidence is needed to validate PCB transformation by the product of this *rdhA*.

### Correlations between Overall MAG Gene Expression within the PCB-Dehalogenating Community

**Figure 5:**
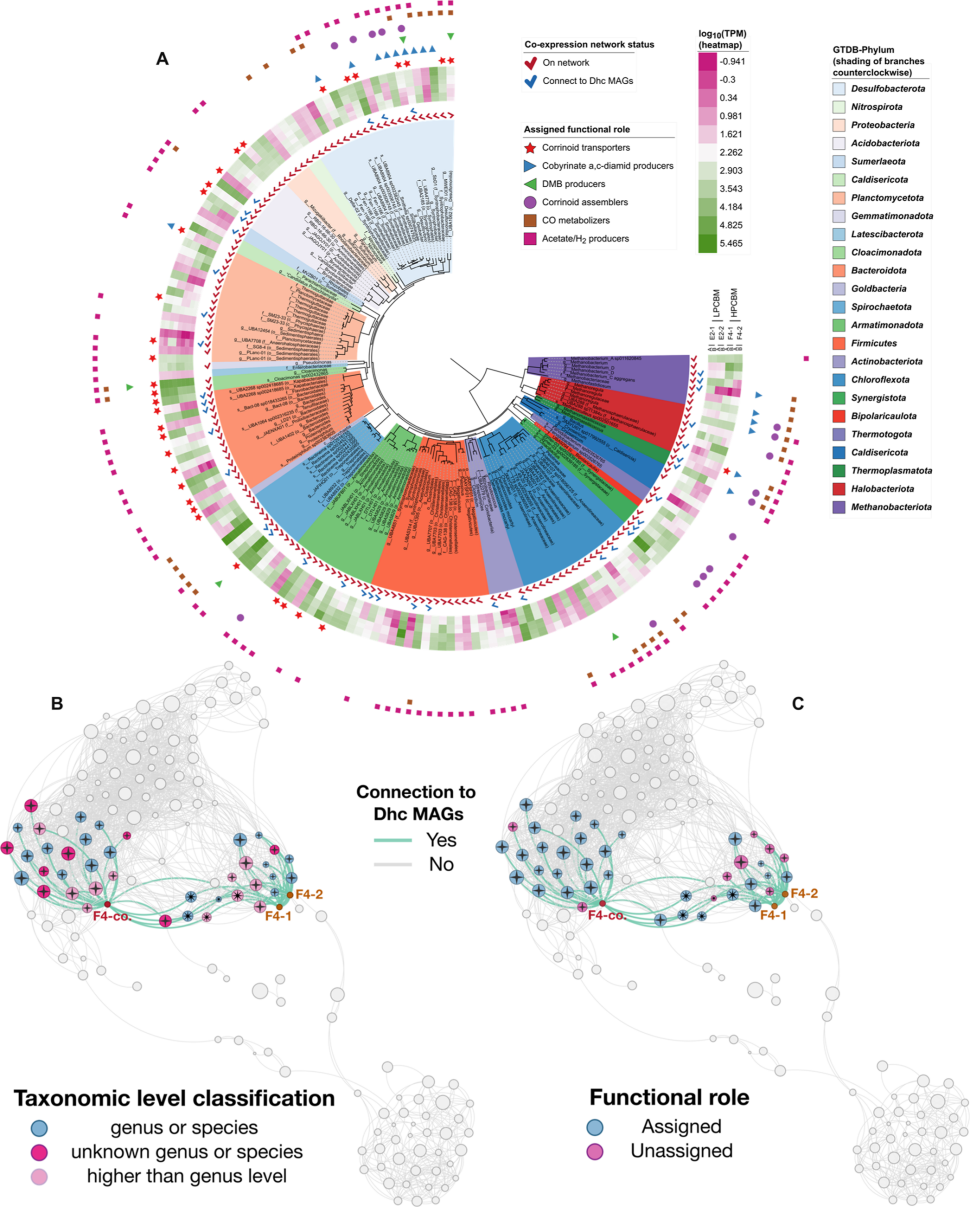
(A) Maximum likelihood phylogenetic tree of MAGs showing phylum classifications, MAG gene expression as log_10_(TPM), assigned functional roles, and status in the coexpression network (i.e., on network, connected to Dhc MAGs). Shaded colors of the branches indicate the phylum. The heatmap shows the log_10_(TPM) of MAGs in the LPCBM and HPCBM metatranscriptomes. Check marks indicate MAGs comprising the network (red) and connected to *Dehalococcoides* MAGs (blue). Assigned functional roles are represented by different markers (corrinoid transporters: red star, cobyrinate diamide producers: blue triangle, DMB producers: green triangle, corrinoid assemblers: purple circle, CO metabolizers: brown square, and acetate/H_2_ producers: pink square). (B) Coexpression network highlighting MAGs connected with *Dehalococcoides* MAGs based on taxonomic classification and (C) based on functional roles (assigned or unknown). Each node shown represents an MAG. Lines between the nodes indicate a significant correlation (i.e., Spearman’s rank correlation coefficient >0.8 and adjusted *p*-value <0.05). Correlation to any *Dehalococcoides* MAGs was in green lines. The node size is proportional to the average log_10_(TPM) in HPCBM metatranscriptomic samples. Nodes representing *Dehalococcoides* MAGs are labeled and are marked in brown. Nodes representing MAGs connected to both coassembled and individually assembled *Dehalococcoides* MAGs are marked with a star sign in the center. Nodes representing MAGs connected to either coassembled or individually assembled *Dehalococcoides* MAGs are marked with a plus sign in the center.

The coexpression network analysis revealed that 154 of 160 MAGs were significantly correlated to others (Figure 5). Of these, 39 MAGs were significantly correlated to *Dehalococcoides* MAGs. We investigated the possibility that these correlations were related to metabolic functions that support *Dehalococcoides* growth.

The growth of *Dehalococcoides* requires hydrogen, acetate, and corrinoid cofactor^8^ that are produced by other microbes in the community. MAGs that express a hydrogenase gene and any short chain fatty acid transformation gene could be considered acetate and hydrogen producers. Microbes that salvage, assemble, and transport portions of the corrinoid cofactor are also beneficial to *Dehalococcoides* growth. Important functions include cobyrinate a,c-diamide production (expression of *cbiA*), 5,6-dimethylbenzimidazole (DMB) production (expression of DMB synthase), corrinoid salvaging (expression of *cobS/cobV*), and corrinoid transport (expression of *btuB* and either *btuC* or *btuD*). The *Dehalococcoides* MAGs contain an incomplete Wood–Ljungdahl pathway (Figure S10), which could lead to carbon monoxide (CO) accumulation and impact growth.^15^ Thus, *Dehalococcoides* could benefit from the activity of CO metabolizers (as identified by the expression of a CO dehydrogenase gene). A gene encoding resuscitation-promoting (RP) factor (*rpfB*) reportedly accelerated the enrichment of an anaerobic PCB-dechlorinating culture,^79^ suggesting that RP-producing microbes, i.e., those expressing *rpfB*, can enhance *Dehalococcoide*s growth. More details of functional roles associated with *Dehalococcoides* growth are described in Section S6.

We examined expression of selected genes involved in the metabolic processes described above within each of the 160 MAGs (Section S6, Figure S11, and Table S7). MAGs were assigned a functional role if they contained and expressed one or more key functional genes in a minimum of two samples. Among the 160 MAGs, 112 were assigned at least one functional role (Figure 5 and Table S7). Of these 112, 31 were significantly correlated to *Dehalococcoides* MAGs. A higher proportion of MAGs correlated with *Dehalococcoides* had a functional role (31/39) compared to that of the MAGs overall (112/160). This is anticipated as those functions were specifically chosen for promoting the growth of *Dehalococcoides*. This is the first time that metabolic functions known to benefit the activity of *Dehalococcoides* were verified in a microbial community with coexpression analysis.

Of the 31 MAGs correlated to *Dehalococcoides* MAGs and assigned at least one role, there were 25 acetate/H_2_ producers (64.1%), 11 corrinoid transporters/competitors (28.2%), 7 CO metabolizers (17.9%), 6 corrinoid assemblers (15.4%), 4 cobyrinate a,c-diamide producers (10.3%), 2 DMB producers (5.1%), and 1 RP producer (2.6%). This is consistent with the total gene expression of those MAGs related to *Dehalococcoides* MAGs (Figure S13) and suggests that DMB could be limiting.

The percentage of the total gene expression related to *Dehalococcoides* MAGs for cobyrinate a,c-diamide (72.2%) and DMB producers (62.4%) were elevated compared to the other roles (13.8–42.4%) (Figure S12), which highlights the importance of these intermediates in supporting growth of *Dehalococcoides*. In contrast to cobyrinate a,c-diamide and DMB producers, gene expression by corrinoid assemblers correlated to *Dehalococcoides* MAGs was 13.8%. The relatively low representation and activity of corrinoid assemblers and DMB producers in this community suggest that corrinoid synthesis processes could limit growth of *Dehalococcoides* and slow PCB dechlorination.

Although RP was reported to accelerate enrichment of a PCB-dechlorinating culture,^79^ RP producers appear to be rare in sediment microbial communities. The relatively high percentage of total gene expression attributed to RP producers correlating to *Dehalococcoides* MAGs (42.4%) suggests that adding the exogenous resuscitation-promoting factor (Rpf) could be an alternative strategy for developing PCB-dechlorinating enrichment cultures.

Specific CO metabolizers and acetate/H_2_ producers appear to be less critical roles supporting *Dehalococcoides* growth because a smaller percentage of their total expression (29.0% for CO metabolizers and 26.1% for acetate/H_2_ producers) was related to *Dehalococcoides* MAGs and their widespread presence (86/154 MAGs). This suggests that *Dehalococcoides* growth in these microcosms was less affected by CO accumulation or carbon source and electron donor limitations.

Interestingly, two MAGs (GCA_021372335.1_ASM2137233v1 and GCA_021372315.1_ASM2137231v1) classified as methanogens (g_*Methanomassiliicoccus*) and identified as cobyrinate a,c-diamide producers and corrinoid assemblers, were also correlated with *Dehalococcoides* MAGs (Table S8), although this genus would compete with *Dehalococcoides* for H_2_.^80^ Indeed, nine MAGs classified as methanogens (g_*Methanoregula*, s_*Methanoculleus* sp002501655, g_*Methanofastidiosum*, f_*Methanobacteriaceae*, and g_*Methanobacterium*) were not significantly coexpressed with *Dehalococcoides* MAGs (i.e., TPM values were 1–2 orders of magnitude higher than *Dehalococcoides* MAG TPMs). The taxonomy of these methanogen MAGs suggest that they utilize H_2_ for methane production.^81−85^ This suggests that network coexpression analysis can reveal potential competition for H_2_ between methanogens and OHRB. Competition for H_2_ could also contribute to slow *Dehalococcoides* growth despite the widespread presence and activity of acetate/H_2_ producers in the community.^86^

Another MAG, classified as *g_Rhodococcus*, harbored several aromatic-ring cleavage dioxygenases and a complete aerobic corrinoid biosynthesis pathway, making it a potential aerobic PCB degrader and corrinoid producer. However, gene expression from this MAG was low (Table S8) and its corrinoid biosynthesis genes were not expressed. Only three of its expressed genes correlated with *Dehalococcoides* (Table S9). A PCB-degrading *Rhodococcus* strain was previously found in a viable but nonculturable (VBNC) state under anaerobic conditions, and its ability to degrade PCBs recovered after exposure to oxygen.^87^ We speculate that this *Rhodococcus* MAG represents an aerobic PCB degrader in a VBNC state under the anaerobic conditions in these microcosms; however, further tests would be needed to validate its VBNC state.

Several MAGs with less characterized taxonomy and without an assigned functional role were also correlated to *Dehalococcoides* MAGs (Figure 5B,C). In previous dechlorinating microbial community studies, co-occurrence networks were based on relative abundance of OTUs/ASVs obtained using 16S rRNA amplicon sequencing.^3,19,20,46−48,88^ Because the functional potential of the OTUs/ASVs from less-studied taxonomies is typically poorly understood, this could lead to overemphasis on better-known taxonomies while overlooking other possibly important taxa. A genome-resolved approach can overcome these phylogeny/function issues. For example, MAG 7_bin.119 classified as o_*Syntrophales* represents a versatile potential CO metabolizer and producer of DMB, corrinoids, H_2_, and acetate that was also significantly correlated with the coassembled *Dehalococcoides* MAGs (Table S8). We also note that the network analysis was performed for one time point, which was 200 days after stimulating the microcosms with an electron donor. A network based on a temporal metatranscriptomics analysis might have revealed additional microbial community interactions that occurred either prior to or after the onset of *Dehalococcoides* growth in the HPCBM.

To further explore possible functional connections between MAGs without assigned functional roles that correlated with the *Dehalococcoides* MAGs (Figure 5C), an additional network was constructed with expressed genes from MAGs without assigned functional roles. An MAG (classified as class *Sumerlaeia*) had a high TPM, which led to correlations between 1011 expressed genes in that MAG to those in the *Dehalococcoides* MAGs (Figure S13). More than half (550) of the genes were annotated as hypothetical proteins. The remaining 461 genes with KO identifiers were used to reconstruct the metabolism pathways. Although many expressed genes were categorized under fundamental cellular processes (e.g., carbon metabolism (27) and biosynthesis of secondary metabolites (73) and amino acids (24)), we also noted 35 expressed genes involved in biofilm formation and 15 expressed bacterial secretion system genes for cell wall-degrading enzymes (Table S9). These results are consistent with the observed expression of a cyclic di-GMP phosphodiesterase gene, which controls bacterial cell adhesion^89^ by *Dehalococcoides* in the HPCBM (Figure 3). Overall, these results suggest that biofilm formation was potentially beneficial to *Dehalococcoides* in the HPCBM.

## Environmental Implications

Genome-resolved metagenomics and metatranscriptomics yielded a relatively large database (160) of representative reference MAGs from indigenous PCB-dechlorinating sediment communities compared to previous genome-resolved analyses of dechlorinating communities^62−64^ and further enabled identification of active novel taxa and expressed functions and potentially new interactions in sediment microbial communities that dechlorinate PCBs. Genome-resolved metatranscriptomics facilitated the detection and analysis of an expressed (and previously unknown) RDase gene in the *Dehalococcoides* MAGs. The evidence presented strongly suggests that *Dehalococcoides* are responsible for anaerobic PCB dechlorination processes in the HPCBM and also likely contribute to natural attenuation of PCBs at the site.^21^ Although 112 MAGs were assigned functions that could support *Dehalococcoides* growth, only 31 were strongly coexpressed with *Dehalococcoides* MAGs. The low proportion (13.8%) of corrinoid producers that correlated to *Dehalococcoides* MAGs could explain the slow growth of *Dehalococcoides* in the microcosms. Future work could focus on shaping the desired microbial profile by adjusting the physicochemical parameters of the microbial habitat to increase the dechlorination rate. Overall, genome-resolved metagenomic and metatranscriptomic analyses provides valuable insights into the ecology and interactions of microbial communities containing OHRB (e.g., *Dehalococcoides*) and could facilitate new research aimed at enhancing reductive dechlorination rates in bioaugmentation cultures and at contaminated sites via biostimulation.

## Data Availability

Raw metagenomic reads (SRA accession numbers SRX11347095 to SRX11347098) and metatranscriptomic sequencing data (SRA accession numbers SRX11347095 to SRX11347098) and high-quality MAGs are available under BioProject accession number PRJNA743546 in NCBI. The scripts used for the metagenomic and metatranscriptomic analysis are accessible via GitHub: github.com/danghongyu/Workflow_for_genome_resolved_analysis.

## Supporting Information Available

Additional information about nucleic acid extraction, qPCR settings, sequencing, and bioinformatics analysis; PCB concentration in microcosms; results for the diversity of recovered MAGs, RDases, and functional role assignment; taxonomic classification, phylogeny, RPKM, covered fraction and functional annotation of the MAGs; oligonucleotide primer information; pertinent qPCR parameters in accordance with MIQE guidelines; comparison of individual PCB congeners as measured in high- and low-PCB-contaminated sediment microcosms; and *Dehalococcides* pangenomic analysis with selected genomes/assemblies from NCBI and *Dehalococcoides* MAGs with a heatmap showing the average nucleotide identity (ANI) (PDF)
List of MAGs with a completion (comp.) ≥80; RPKM and GTDB-tk taxonomy classification of dereplicated MAGs and the correlated NCBI phylum and genus; pairwise BLAST results of genes in the same cluster with identified PCBs dechlorinating genes from *Dehalococcoides* pangenomic analysis; all expressed genes from individually assembled and coassembled *Dehalococcoides* MAGs; functional role assignment for MAGs based on the expression of genes involved in corrinoid synthesis, lactate and H_2_ production, CO metabolism, and RP production; correlation to *Dehalococcoides* MAGs and functional role assignment for all MAGs presented in the network, together with log_10_(TPM), lowest taxonomy assignment, and phylum; and metabolism reconstruction of significantly expressed genes from MAG 5_191 and genes from the MAG of *Rhodococcus* (XLSX)
