# Supplementary material for: Genome-Resolved Metagenomics and Metatranscriptomics Reveal Insights into the Ecology and Metabolism of Anaerobic Microbial Communities in PCB-Contaminated Sediments
Source: Environ Sci Technol. 2023 Oct 19;57(43):16386–98. doi: 10.1021/acs.est.3c05439 (PMC10621002; doi:10.1021/acs.est.3c05439)
Supplement: Supplementary file 1 — es3c05439_si_001.pdf [file es3c05439_si_001.pdf]

Genome-resolved metagenomics and metatranscriptomics reveals insights into the ecology and metabolism of anaerobic microbial communities in PCB-contaminated sediments

Authors:

Hongyu Dang, Jessica M Ewald, Timothy E Mattes[1]*

[1]Department of Civil and Environmental Engineering, 4105 Seamans Center, University of Iowa, Iowa City, IA, 52242, USA

*Corresponding author:

Fax: (319) 335-5660

Email: tim-mattes@uiowa.edu

This supplemental information document includes:

Supplementary methods (Section S1-S3), Supplemental Results/Discussion (Section S4-S6), Figures S1-S13, Tables S1 and S2, and Tables S3-S9 (Provided in a separate Excel file)

S1. Supplemental methods

RAMM composition

The following was added to 1 L of RAMM,: 0.27 g KH_2PO_4 and 0.35 g K_2HPO_4 (phosphate buffer, adjusted to pH 7.0); 0.53 g NH_4Cl, 75 mg $CaCl_2 \cdot 2H_2O$, 100 mg $MgCl \cdot 6H_2O$, and 20 mg $FeCl_2 \cdot 4H_2O$ (mineral salts);0.5 mg $MnCl_2 \cdot 4H_2O$, 0.05 mg H_3BO_3, 0.05 mg $ZnCl_2$, 0.03 mg $CuCl_2$, 0.01 mg $NaMo_4$ $2H_2O$, 0.5 mg $CoCl_2 \cdot 6H_2O$, 0.05 mg $NiCl_2$ $6H_2O$, and 0.05 mg Na_2SeO_3 (trace metals); and 1.2g $NaHCO_3$ (bicarbonate).

DNA and RNA extractions, qPCR and sequencing

DNA and RNA extraction procedures were described previously.[1] DNA was extracted from withdrawn sediment slurry (2 mL) using a modified version of the DNeasy PowerWater Sterivex Kit protocol.[2] DNA concentrations were measured with the Qubit 4 fluorometer using dsDNA high sensitivity assay kit reagents (Thermofisher Scientific, Waltham, MA). RNA was extracted from duplicate slurry samples (5 mL; total eight RNA samples) from each biological replicate with the RNeasy Powersoil Total RNA Kit to increase statistical power. The RNA extracts were then treated with DNase using the TURBO DNA-Free Kit (Thermofisher Scientific, Waltham, MA) and purified with the Zymo Direct-zol RNA MiniPrep Plus kit (Zymo Research Corporation, Irvine, CA).

DNA samples for sequencing were sheared with the Covaris E220 ultrasonicator (Covaris, Inc., Woburn, MA, USA) and indexed with the Roche Kapa HyperPrep kit. RNA samples were indexed with the Illumina Stranded Total RNA Prep with Ribo-Zero Plus, with the addition of supplemental probes to increase removal of Archaeal rRNA.[1] Both DNA and RNA

libraries were sequenced on Illumina NovaSeq 6000 flow cells (2×150-bp paired-end reads) at the University of Iowa Institute of Human Genomics (IIHG; Iowa City, IA, USA).

To observe *Dehalococcoides* growth over time, quantitative PCR (qPCR) was conducted with DNA collected over time to estimate the abundance of *Dehalococcoides* 16S rRNA genes.[3] Briefly, for each set of primers (Table S1), a standard curve that ranged from 3×10^7 gene copies to 30 gene copies was prepared in triplicate to estimate the abundance of the target sequences. Statistical differences for qPCR data between from HPCBM and LPCBM were analyzed with an independent two-sided t-test with $\alpha = 0.05$. Additional qPCR parameters are provided in Table S2 according to MIQE guidelines.[4] Sequences amplified using DNA from the lagoon sediment as a template and transformed into clone libraries served as the standards for qPCR enumeration of *Dehalococcoides* 16S rRNA sequences.[5]

S2. Taxonomic classification, phylogeny, RPKM, covered fraction and functional annotation of the MAGs

MAG taxonomy was determined using GTDB-Tk (version 2.1.1)[6] with classify workflow (classify_wf) and the database release R207_v2. GTDB-Tk dependencies included Prodigal (version 2.6.3),[7] HMMER (version 3.1b2),[8] pplacer (version v1.1.alpha19),[9] FastANI (version 1.3),[10] FastTree (version 2.1.11).[11]

Inferred protein sequences of single copy marker genes in MAGs, as retrieved by CheckM, were concatenated and then aligned with Clustal Omega (version 1.2.3).[12] The aligned file was used to build a phylogenetic tree using IQ-TREE (version 2.0.3) with ModelFinder Plus flag and ultrafast bootstrap number 1000.[13] LG+R10 was the selected best-fit model. The tree was then visualized in iTOL.[14]

Reads per kilo base per million mapped reads (RPKM) and coverage fraction of the MAGs were determined with coverM (version 0.4.0) (https://github.com/wwood/CoverM) using default settings. Functional genes in the MAGs were annotated with Prokka (version 1.14.6) using default settings.[15] The genes were also annotated by running a hmmsearch against KOfam, a customized HMM database of KEGG orthologs (KOs),[16, 17] using HMMER (version 3.1b2),[8] with the program "anvi-run-kegg-kofams" of Anvi'o. The KOs of annotated genes from each MAG were uploaded to the webpage Reconstruct tool (https://www.genome.jp/kegg/mapper/reconstruct.html) of KEGG mapper to reconstruct metabolic pathways.[18]

***Dehalococcoides* pangenomic analysis**

The pangenomic analysis followed the Anvi'o pangenome workflow.[19] All *Dehalococcoides* genomes available in NCBI (51, including the co-assembled *Dehalococcoides* MAGs) were used. The program "anvi-gen-contigs-database" and "anvi-import-functions" were used to store downloaded genomes and individually assembled *Dehalococcoides* MAGs as contig databases and import the functions of identified coding regions. Genome storage for all contig databases was performed with "anvi-gen-genomes-storage". The final pangenomic analysis was performed with "anvi-pan-genome". The program anvi-compute-genome-similarity with PyANI (version 0.2.12) was used to compute average nucleotide identity (ANI) across genomes and MAGs.[20] The pangenome was visualized with "anvi-display-pan".

A smaller pangenomic analysis of *Dehalococcoides* MAGs and genomes with an ANI >95% with the *Dehalococcoides* MAGs was conducted with a similar procedure as described above. The difference was that inferred amino acid sequences of single-copy genes across the

genome were retrieved with the program "anvi-get-sequences-for-gene-clusters" with parameters "--min-num-genomes-gene-cluster-occurs 48 --max-num-genes-from-each-genome 1 --concatenate-gene-clusters". IQ-TREE (version 2.0.3) was used to build the phylogenetic tree with ModelFinder Plus flag and ultrafast bootstrap number 1000.[13] JTT+F+R2 was selected as the best-fit model. The program "anvi-import-misc-data" was used to import the tree result to the pangenomic analysis.

S3. Comparison and phylogeny of reductive dehalogenase genes in *Dehalococcoides* MAGs and genomes

Among the validated PCB-dechlorinating bacteria, only *Dehalococcoides* strain CG4 had >95% ANI to the individually assembled *Dehalococcoides* MAGs described here. The MAGs were annotated with Prokka (version 1.14.6) [15] using the functions of *D.* CG4 as reference. Inferred amino acid sequences of reductive dehalogenase (RDase) genes in MAGs, annotated using strain CG4 functions and the default Prokka database,[15] were aligned with RDase sequences retrieved from the contaminated site by cloning [3, 5] using BLAST plus (version 2.13.0).[21] Sequences with >90% identity were denoted as the same for comparing RDase genes found in MAGs and clone libraries. The expressed RDase genes from *Dehalococcoides* MAGs were also submitted to Pfam to identified the conserved domain.[22]

RDase amino acid sequences (1040 sequences) were collected from the Reductive Dehalogenase Database (RDase DB) (accessed on 03/28/2023).[23] All RDase sequences from MAGs, clone libraries and RDase DB were aligned with Clustal Omega (version 1.2.3).[12] A phylogenetic tree was constructed with IQ-TREE (version 2.0.3) using the ModelFinder Plus flag

and ultrafast bootstrap number 1000.[13] LG+F+R9 was selected as the best-fit model. The tree was visualized in iTOL.[14]

The tertiary structures of selected *rdhA* products were predicted with AlphaFold2 by using Colabfold2 (version 1.5.2) webserver[24] or downloaded from Pfam.[22] The structures were then visualized in ChimeraX (version 1.6.1).[25]

Table S1. Oligonucleotide primer information.

Target gene	Primer	Sequences (5'- 3')	Product size (bp)	Reference
Dehalococcoides-like 16S rRNA gene (qPCR)	dhc 793F	GGGAGTATCGACCCTCTCTG	193	[26]
	dhc 946F	CGTTYCCCTTTCRGTTCACT		

Table S2. Pertinent qPCR parameters in accordance with MIQE guidelines.

Target gene	Primer concentration (μM)	DNA template mass (ng)	qPCR linear range (gene copies/reaction)	qPCR efficiency	Y-intercept
Dehalococcoides-like 16S rRNA	0.8	10	$30 - 30 \times 10^7$	111.623%	32.38

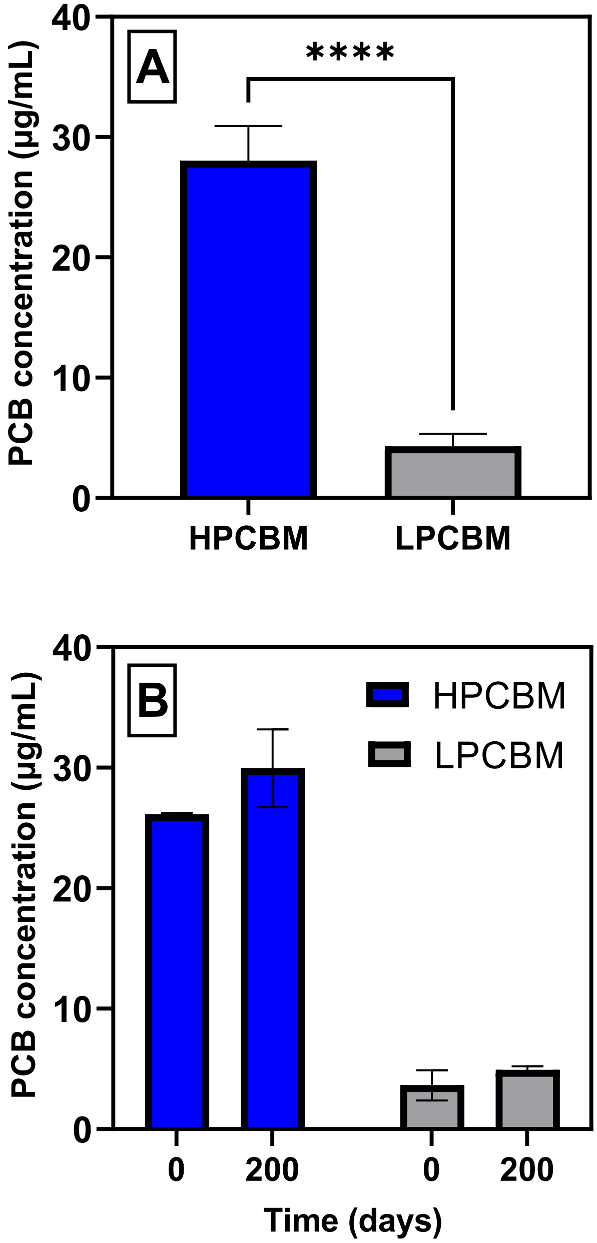

Figure S1. A) Comparison of PCB concentrations between microcosms established with different sediments from the same field site. "****" denotes p value< 0.0001. B) Concentration (µg/mL) of PCBs measured at day 0 and day 200 in microcosm sediment slurries. Error bars represent the standard deviation of duplicate measurements collected from biological replicates of the sediment microcosms.

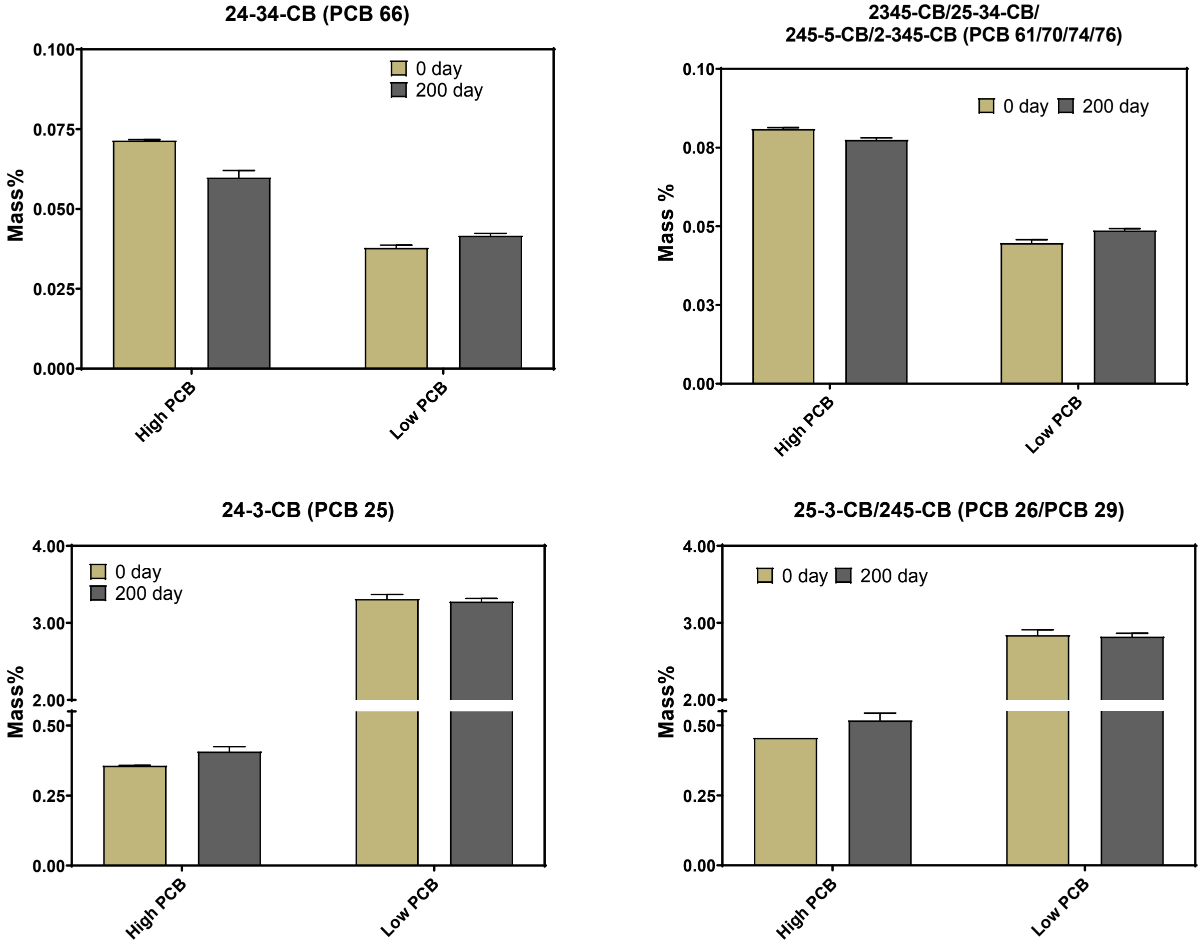

Figure S2. Comparison of individual PCB congeners as measured in high and low PCB contaminated sediment microcosms. There were significant decreases in PCB66 ($p = 0.0182$) and PCB61/70/74/66 ($p=0.0235$) in high PCB microcosms (HPCBM), while there were slight increases in these congeners in the low PCB microcosms (LPCBM). Mass fractions of expected dechlorination products PCB25 and PCB26/29 increased in the HPCBM but did not increase in LPCBM. However, these changes were not statistically significant ($p>0.05$).

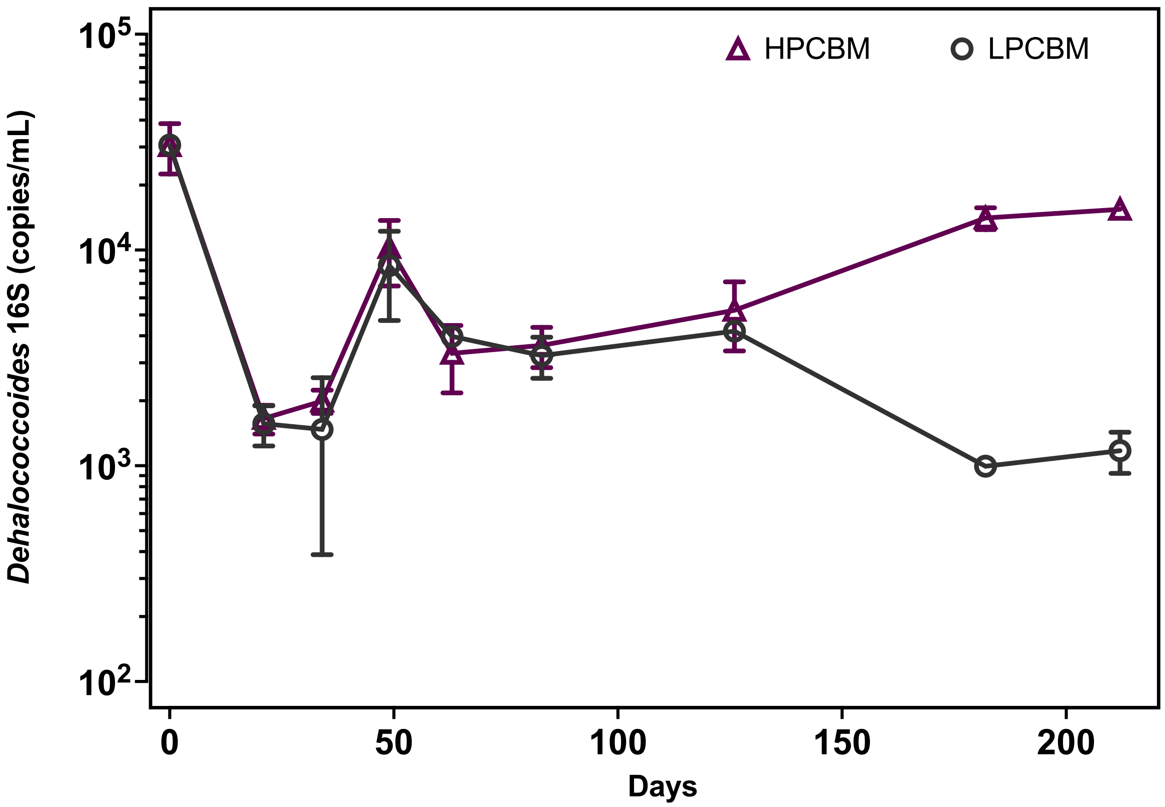

Figure S3. Abundance of *Dehalococcoides* 16S rRNA genes in microcosms over 211 days. Error bars represent variability of duplicate measurements collected from biological replicates.

S4. Dominant phylotypes of recovered MAGs

The phylum *Candidatus* Cloacimonetes, which is commonly present in wastewater systems,[27] was abundant only in LPCBM (Figure S4). In HPCBM, the second most abundant phylum was *Caldiserica*. In LCPBM, this phylum was less abundant than *Chloroflexi* and *Candidatus* Cloacimonetes. The taxonomy of the same metagenomic datasets have been classified with a reads-based approach by using Kraken2 with NCBI taxonomy.[1, 28] A correlation between GTDB taxonomy and NCBI taxonomy for each MAG (Table S4) is used to compare phylum level abundances between the previous reads-based approach and genome-resolved approach in this work. *Euryarchaeota* were the most abundant by both approaches. *Actinobacteria* was dominant based on the reads-based approach. There were 5 *Actinobacteria* MAGs recovered (Figure 2), but it was not among the top 10 most abundant phyla in the MAGs. No MAGs were recovered from the NCBI phyla of *Proteobacteria* and *Bacteroidetes* although they were dominant using the reads-based approach. The main reason for this apparent discrepancy stems from differences between GTDB and NCBI taxonomy designations (Table S4). The MAGs classified as *Proteobacteria* from GTDB were classified *Euryarchaeota* from NCBI. *Bacteroidetes* is not a GTDB phylum. Another potential reason for the difference would be that *Proteobacteria* hosted the most genomes (Table S4), the short reads that were not able to assemble into contigs had a higher chance to match sequences from *Proteobacteria*, which would lead to a bias that *Proteobacteria* was dominant in a microbial community.

Considering taxonomic levels beyond phylum, the most abundant phylotypes according to RPKM across the metagenomic samples is g_*Cryosericum*, followed by s_*Methanobacterium aggregans*, and three other MAGs classified as g_*UBA6107* (f_*Anaerolineaceae*), s_*UBA1064* sp002316235 (f_*Tenuifilaceae*) and g_*Bact-08* (o_*Bacteroidales*). The other abundant

phylotypes based on average $\log_{10}$ (RPKM) in HPCBM samples are s_*Smithella* sp000747625, s_*Methanobacterium* sp011620845, f_*Methanobacteriaceae* and g_*JAFIHQ01* (o_*Treponematales*). Several abundant MAGs could only be classified at the family level or higher, including f_*MVZB01* (c_*Sumerlaeia*), s_*UBA6107* sp018056725 (f_*Anaerolineaceae*), s_*Syner-03* sp002839185 (f_*Synergistaceae*) and s_*UBA8904* sp002382045 (f_*Smithellaceae*).

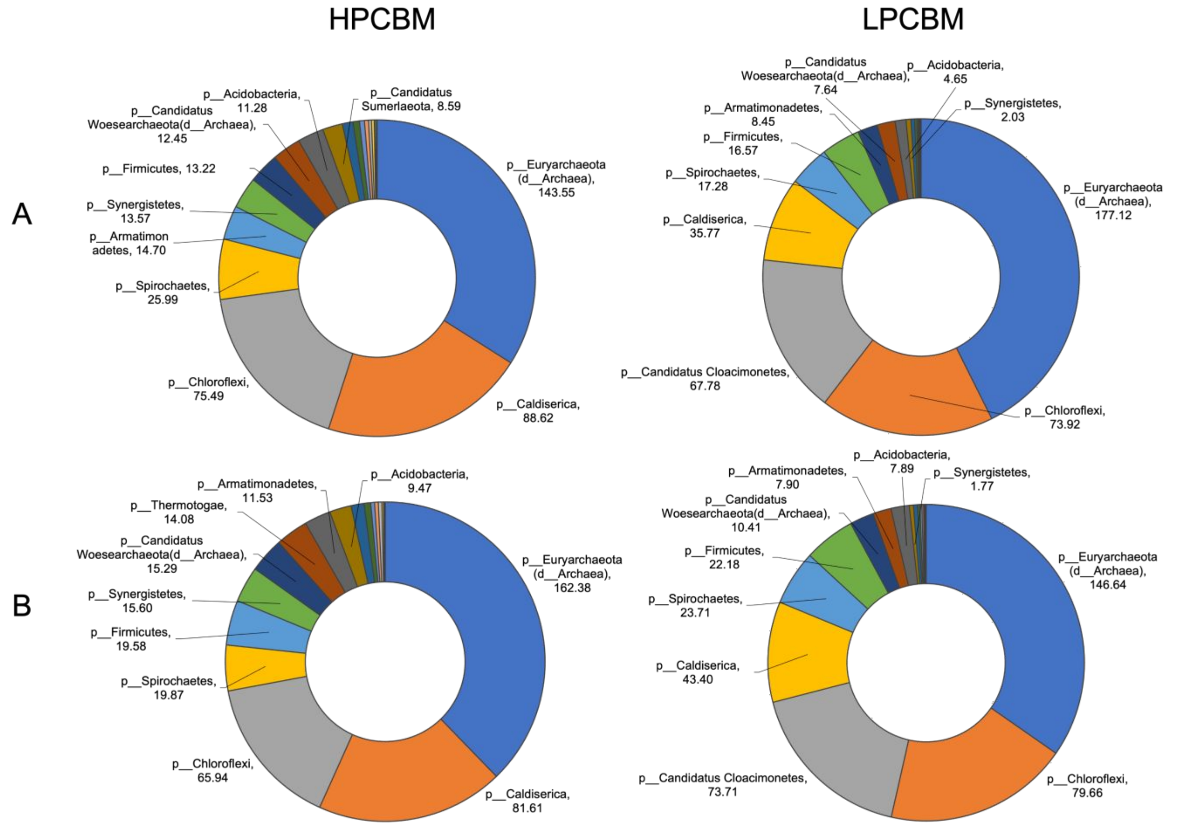

Figure S4. Relative abundance of recovered MAGs at phylum level in the duplicate metagenome samples of (A) HPCBM (F4_1, F4_2) and (B) LPCBM (E2_1, E2_2). The top 10 most abundant phyla are labeled and shown along with their RPKM values.

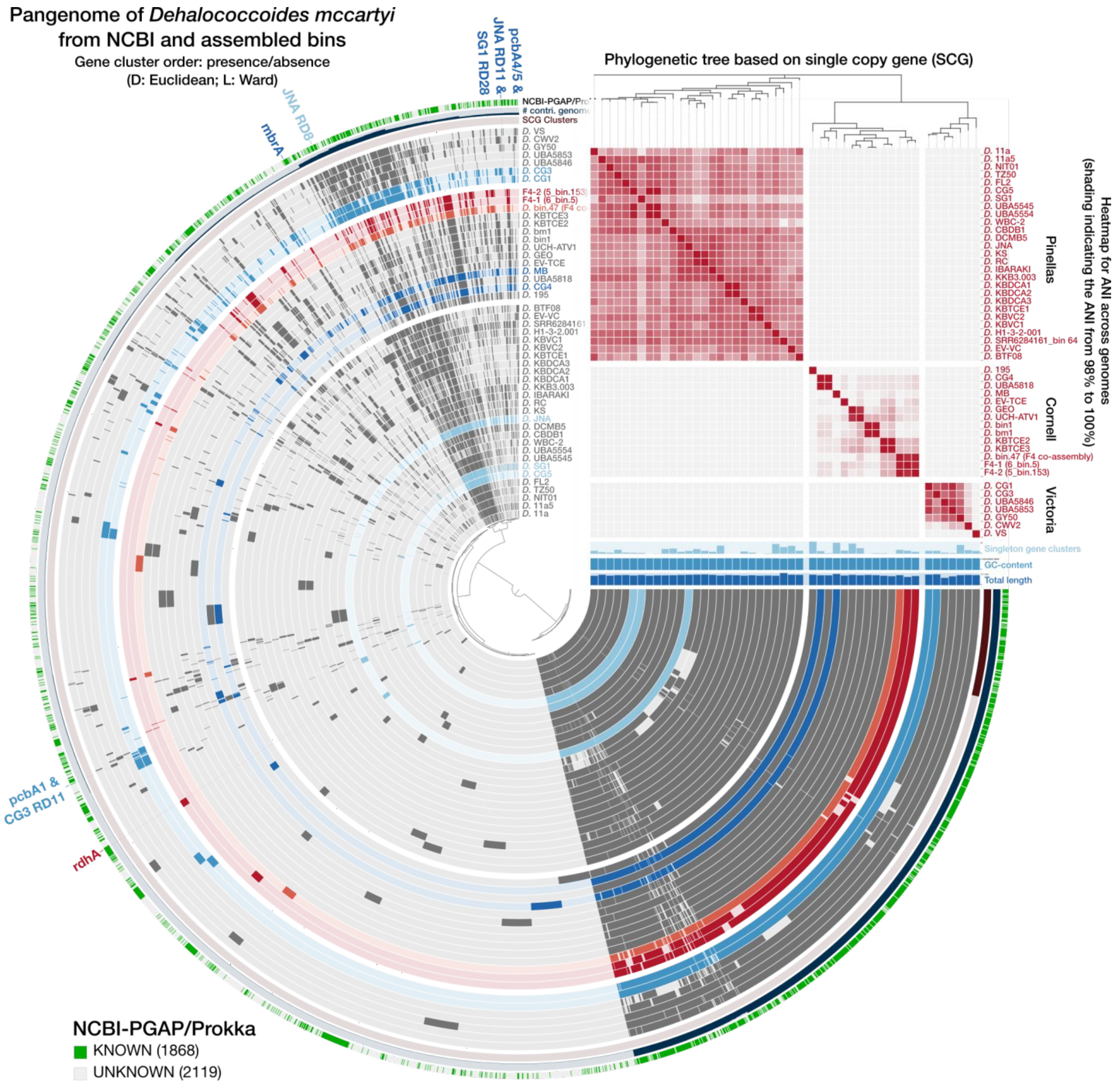

Figure S5. *Dehalococcides* pangenomic analysis with selected genomes/assemblies from NCBI and *Dehalococcoides* MAGs with a heatmap showing the average nucleotide identity (ANI). The dendrogram in the center displays the hierarchical clustering of genes based on their sequence composition and distribution (presence/absence) across genomes and MAGs with Ward's method of Euclidean distance. Dark shading on each genome or MAG ring (from *D*. VS to *D*. 11a) represents regions present in at least one genome/MAG. Light shading on each genome or MAG ring indicates a gap in that region. Colors highlight genomes of known PCB-dechlorinators (blue), co-assembled MAGs (orange), and individually assembled MAGs (red). Other *Dehalococcoides* genomes/assemblies are shaded in grey. The distribution of single copy genes (SCGs) among the genomes and MAGs are indicated in the SCG clusters ring (brown) and were used to build the SCG phylogenetic tree. The contributed (# contri.) genomes ring sums genomes/assemblies containing each gene in the analysis (darker blue). Genes annotated by NCBI-PGAP or Prokka (1868) are indicated in the outer ring (green), with genes of unknown

function (2119), and those encoding tRNAs or rRNAs are indicated in grey. Identified RDase gene clusters for PCB degradation are labeled. The expressed dehalogenase gene from the F4-2 (5_bin.153) MAG is labeled with *rdhA*. The number of singleton gene clusters, GC content and total length of *Dehalococcides* genomes and assemblies are depicted as blue bars under the heat map (from light to dark).

Section S5. In silico examination of identified PCB dehalogenase genes in *Dehalococcoides* genomes

The accession ID of RD11 from CG3 and RD28 from SG-1 were not specified.[29] From the pangenomic analysis, the hypothetical protein (APH13028.1) was identified as RD11, because it had only one amino acid difference with *pcbA1*[29] (Table S5). The functions from assemblies of SG-1 (BioProject: PRJNA256899) were projected from the coding regions annotated by NCBI Reference Sequence. The RD28 was identified to be the gene with locus tag: JD74_RS00015 from NZ_JPRE01000001.1, because it had 5 and 18 amino acid differences with *pcbA4* and *pcbA5*, respectively [29] (Table S5).

Only one gene (*pbrA2*) from polybrominated diphenyl ether degrader *D.* GY50 clustered with *pcbA1* and CG3-RD11. All the regions of CG3-RD11 are present in *pbrA2*, which implied *D.* GY50 could dechlorinate PCBs. In a previous study, *pbrA2* was reported to possess >99% amino acid identity with *pcbA1* and a gene from strain CG3.[30] Two years later, CG3-RD11 was reported, and was only compared with *pcbA1*[29] and not *pbrA2*. As expected, *pcbA4*, *pcbA5*, JNA-RD11 and SG1-RD28 shared more than 95% amino acid identity with each other, which had been reported previously.[29, 31] *D.* 195, *D.* KBVC1, *D.* 11a5 and an assembly SRR6284161_bin.64 also harbored genes with more than 95% amino acid identity to *pcbA4*, *pcbA5*, JNA-RD11 and SG1-RD28. The assemblies: UBA5554, UBA5545 and UBA5818 were not discussed although they also harbored similar genes to *pcbA4*, *pcbA5*, JNA-RD11 and SG1-RD28. It was because those assemblies were reconstructed from metagenomics data in NCBI [32]

rather submitted for new isolates/culture of *Dehalococcoides*. Genes that shared more than 95% amino acid identity to JNA-RD8 were found in other 20 genomes and assemblies of *Dehalococcoides*, which disclosed a wider spectrum for potential PCB degraders than before. Previous work only reported JNA-RD8 shared 94% amino acid identity with a gene from *D.* 195.[31] The genes that shared more than 98% amino acid identity with *mbrA* were present in *D.* DCMB5, *D.* CBDB1 (*cbrA*, CAI82340.1), *D.* KKB3.003, *D.* H1-3-2.001 and an assembly SRR6284161_bin.64. The similarity between *mbrA* and *cbrA* had previously been revealed by a phylogenetic tree.[33] Except the gene cluster for *pcbA1* and CG3-RD11, the other clusters for identified PCB dehalogenase genes were present across different subgroups, which was inconsistent with the observation that *Dehalococcoides* of the same subgroup tended to share similar RDases.[34]

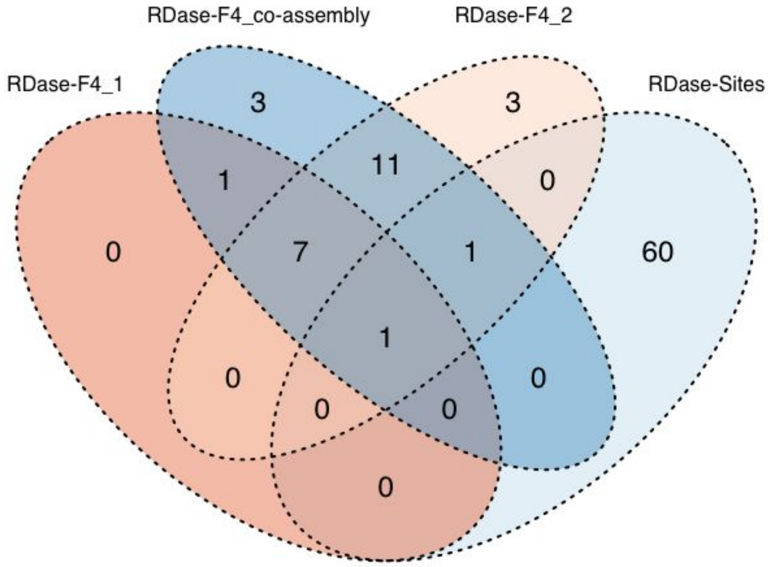

Figure S6. The number of reductive dehalogenases with more than 90% identity among MAGs and PCB contaminated lagoon.

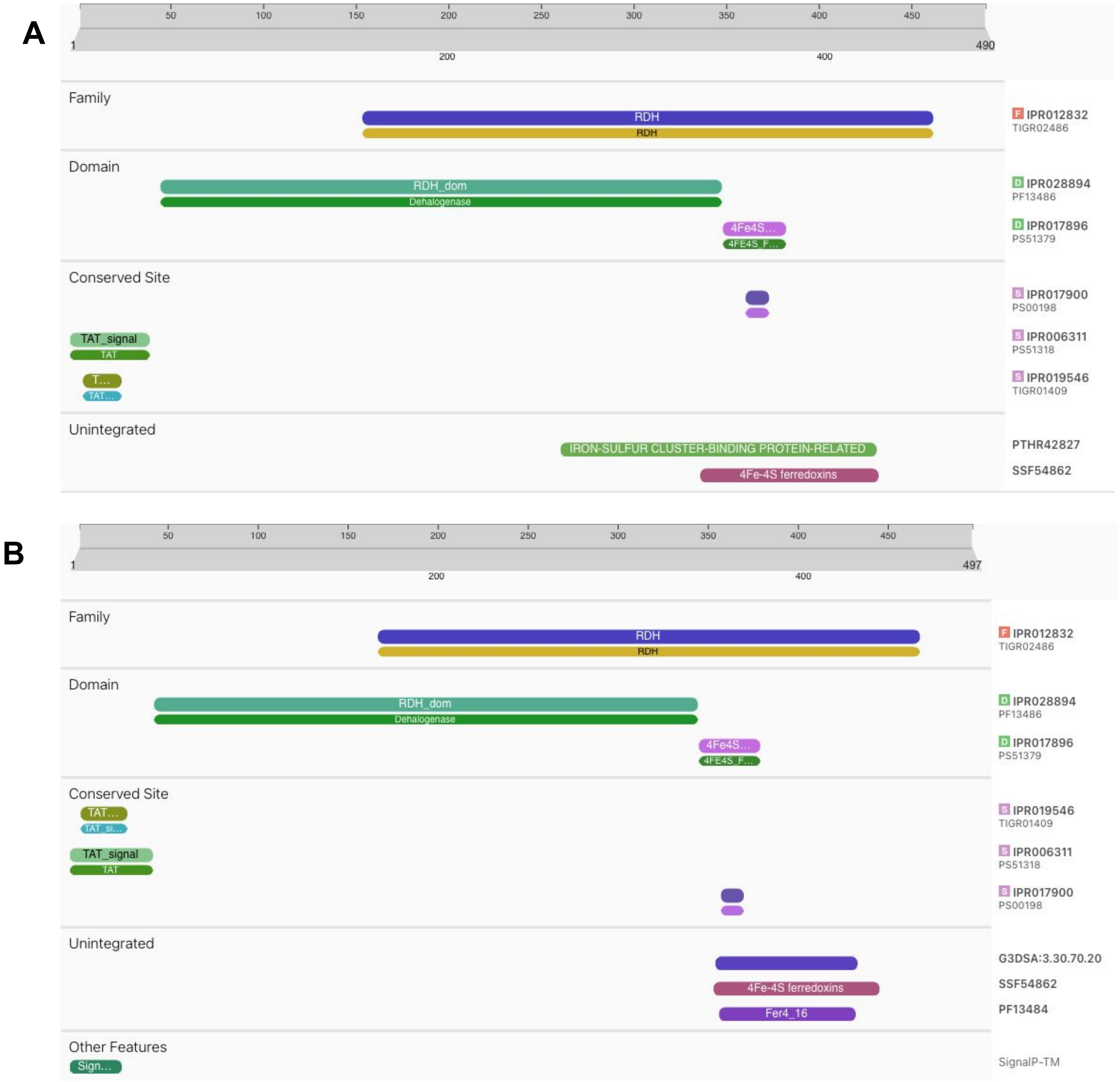

Figure S7. Identification of conserved domain of reductive dehalogenase genes JHMAAFGB_00007 (A) and JHMAAFGB_00005 (B) with Pfam database.

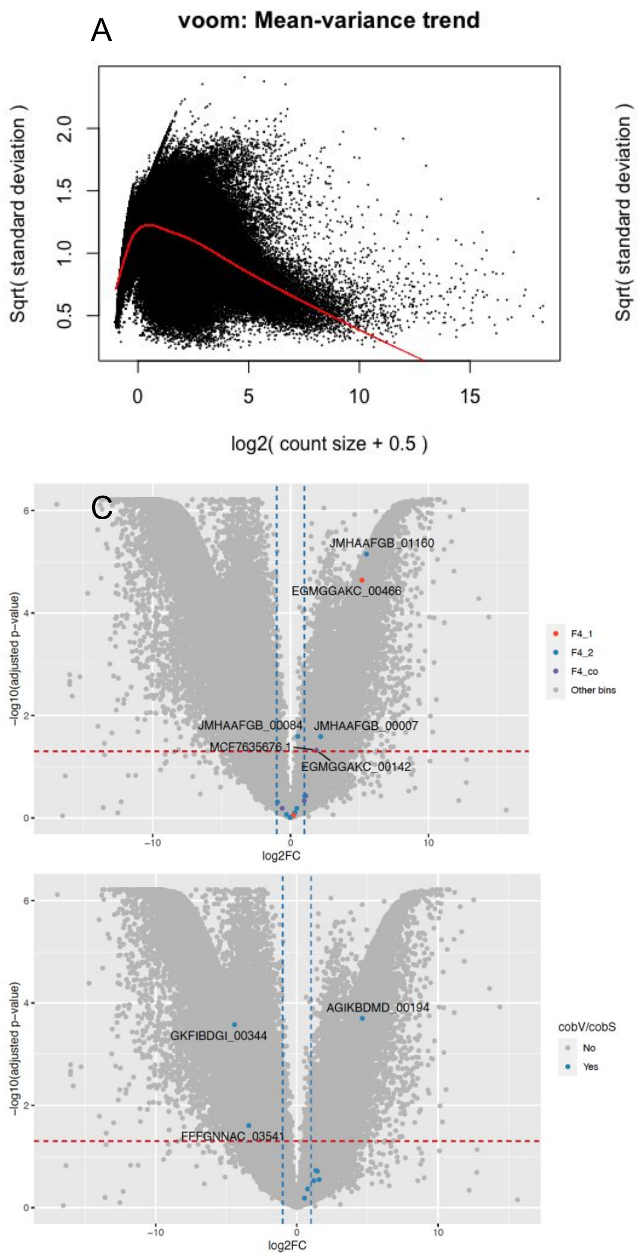

Figure S8. Voom plot of square root residual standard deviations of log2 fold changes against average log2 count (A) before filtering and (B) after filtering genes expressed in at least three samples with a maximum count > 3. (C) Differential expression of highlighted genes from *Dehalococcides* MAGs and (D) for corrinoid synthesis based on the comparison between high (treatment) and low (control) PCB samples. The genes examined with significant differences are labeled with identifiers.

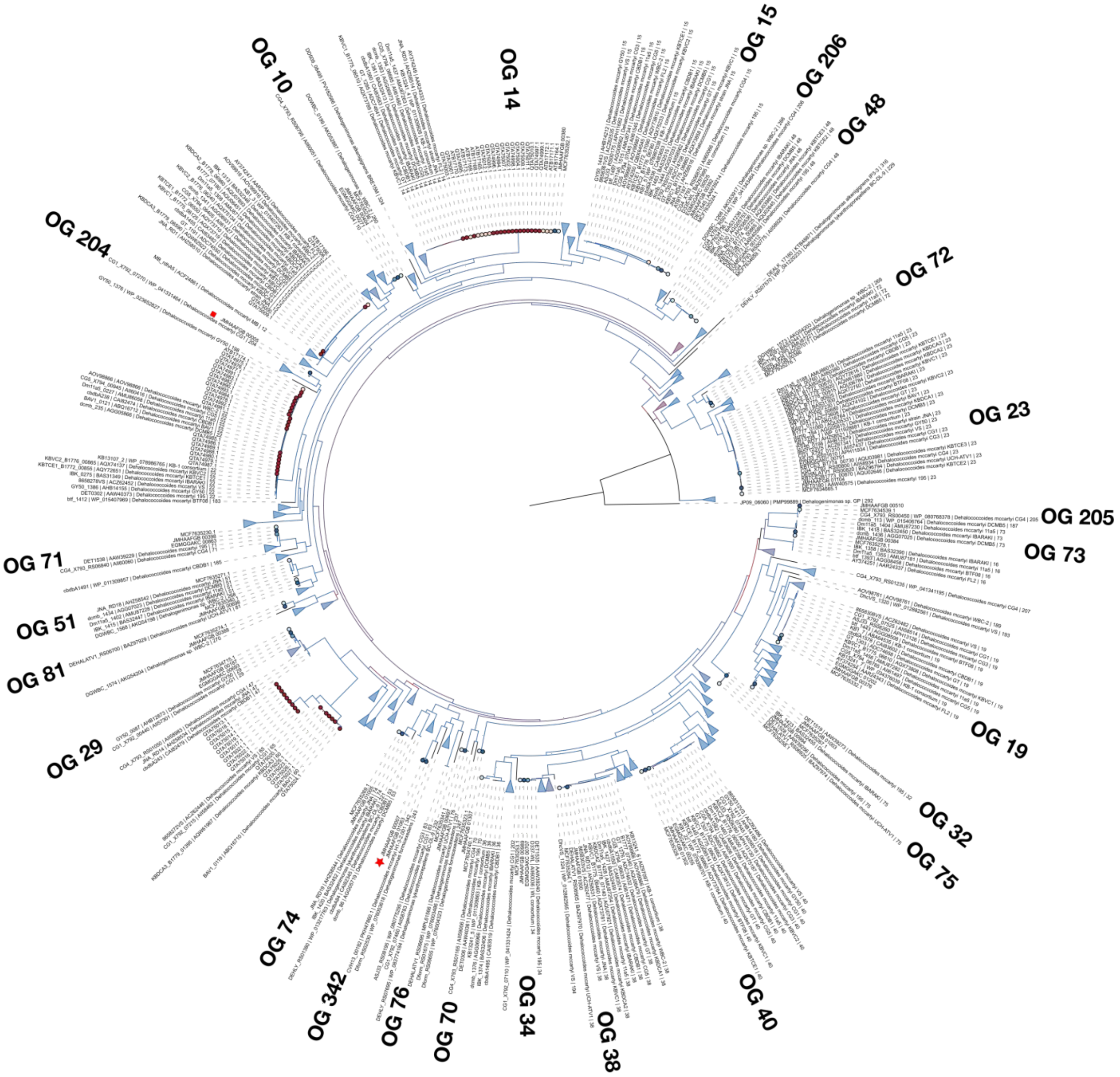

Figure S9. Maximum likelihood phylogenetic tree of reductive dehalogenase genes from PCB-contaminated lagoon sediments (light red circle),[5] microcosms developed from PCB-contaminated lagoon sediments (dark red circle),[3] co-assembled *Dehalococcoides* MAGs (light blue circle)[35] and individually assembled *Dehalococcoides* MAGs (dark blue circle) along with all RDase genes from RDD (branches with no circles). The expressed reductive dehalogenase gene from MAGs in metatranscriptome data is marked with red star (JHMAAFGB_00007) and red square (JHMAAFGB_00005). The branch labels of reductive dehalogenase genes from *Dehalococcoides* strains and KB-1 consortium are shown with the locus identifier, protein accession ID, strain/isolate name and ortholog group (OG) number. Branches containing no reductive dehalogenase genes from the lagoon, microcosms and MAGs are collapsed, shown with a triangle at the end of the branch. The color gradient of the branches represents range of bootstrap values from 30 (red) to 100 (blue). The nearest RDase OGs from *Dehalococcoides* MAGs are labeled.

Section S6. Assigning functional roles that support growth of *Dehalococcoide*s to MAGs in the PCB-dehalogenating community.

Dehalococcoides strains rely on other members of the microbial community to supply hydrogen as electron donor and acetate for biosynthesis. Acetate and hydrogen were produced concomitantly in the metabolism of lactate, pyruvate, propionate and butyrate.[36, 37] Expression of hydrogenases (Hyd, [Fe] hydrogenase; Hyn1, [Ni-Fe] hydrogenase; periplasmic hydrogenase) account for the production of gaseous hydrogen. Acetate CoA-transferase subunit alpha and Butyryl-CoA:acetate CoA-transferase (transform butyrate), lactate dehydrogenase (transform lactate), pyruvate ferredoxin oxidoreductase, formate C-acetyltransferase, pyruvate formate lyase (transform pyruvate) together with hydrogenase provide the potential for producing acetate and hydrogen, which provided the carbon source and direct electron donor for *Dehalococcoides.*

Dehalococcoides growth is also dependent on RDase corrinoid cofactor production[38] by other microbial community members. Reconstructing corrinoid biosynthesis pathways in *Dehalococcoides* MAGs (Figure S10A) revealed the inability for *de novo* corrinoid biosynthesis, which is consistent with previous studies.[39, 40] However, the *Dhc* MAGs harbored genes for corrinoid salvaging when provided with intermediates (e.g., *cobA* to transform cobyrinate a,c-diamid/cobinamide and *cobU*/*cobT* to transform 5,6-Dimethylbenzimidazole (DMB)) along with genes for corrinoid transport (*btuB*, *btuC* and *btuD*). Thus, microbes that can synthesize cobyrinate a,c-diamid/cobinamide (using *cbiA*) and DMB (using DMB synthase) are potentially important for *Dehalococcoides.* Expression of *btuB* is required when assigning the corrinoid transporter role but will miss monoderm (i.e., gram-positive) microorganisms, because *btuB* mediates corrinoid transport across the outer membrane and corrinoid accumulation in periplasm.[41]

Two Wood-Ljungdahl pathway genes, *mteF* and *acsE*, encoding proteins that sequentially reduce 5, 10-methylene-tetrahydrofolate (CH_2-THF) to 5-methyl-tetrahydrofolate (CH_3-THF), and to tetrahydrofolate (THF), were missing in the *Dehalococcoides* MAGs (Figure S11). In other *Dehalococcoides* strains, the incomplete pathway led to carbon monoxide (CO) accumulation which impacted growth.[42] A gene encoding resuscitation-promoting (RP) factor (*rpfB*), reported to accelerate growth of an anaerobic PCB-dechlorinating culture,[43] was also noted in the microbial community.

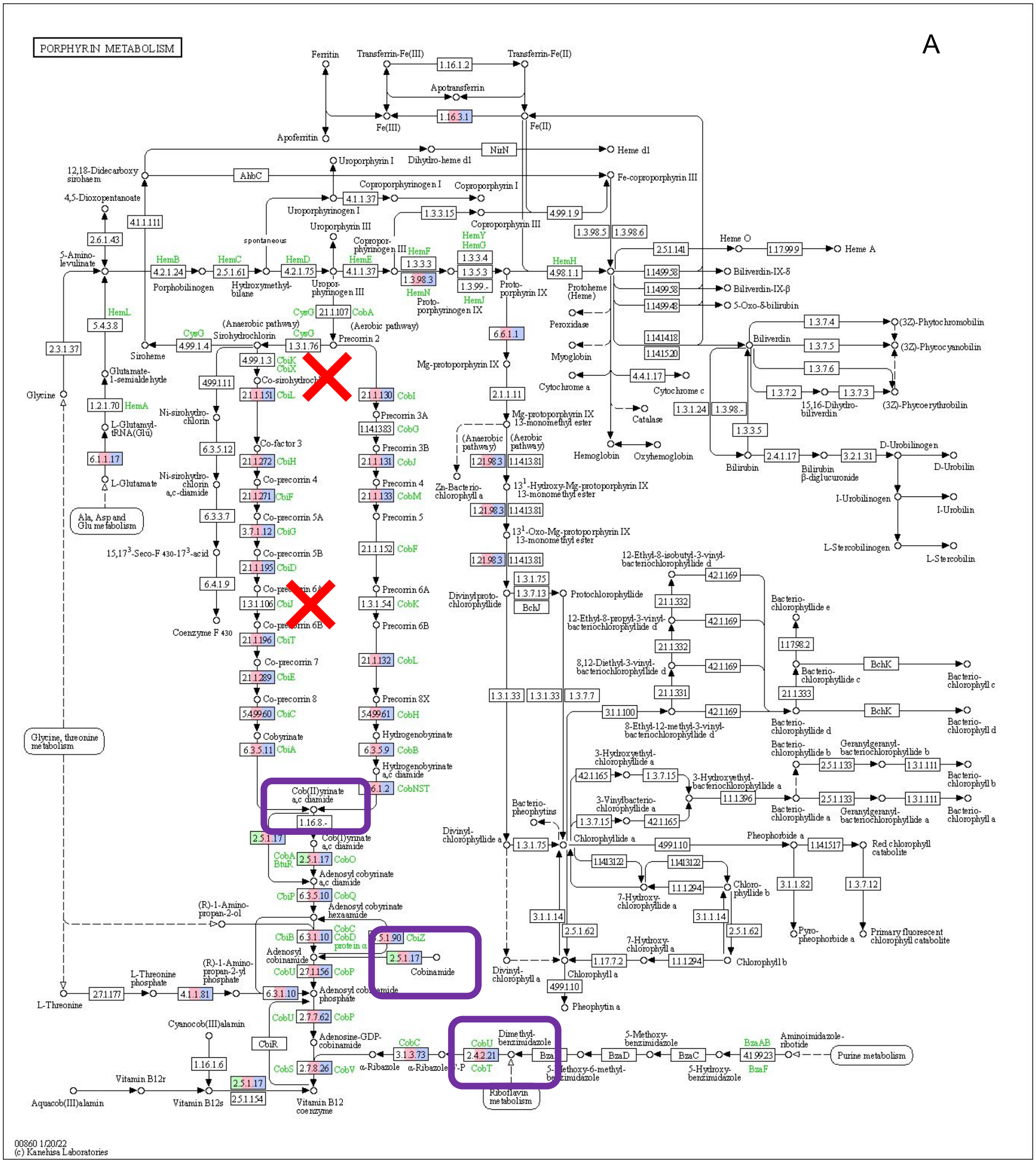
PORPHYRIN METABOLISM
A
Ferritin
Transferrin-Fe(III)
Transferrin-Fe(II)
Apotransferrin
Fe(III)
Fe(II)
Apoferritin
NirN
Heme d1
Dihydro-heme d1
Uroporphyrin I
12,18-Didecarboxy siroheam
AhbC
Fe-coproporphyrin III
4,5-Dioxopentanoate
Coproporphyrinogen I
Coproporphyrin I
Uroporphyrinogen I
Coproporphyrin III
Uroporphyrin III
spontaneous
5-Amino-levulinate
HemB
HemC
HemD
HemE
HemF
HemY
HemG
HemH
HemN
HemJ
HemL
HemA
Porphobilinogen
Hydroxymethyl-bilane
Uropor-phyrinogen III
Copropor-phyrinogen III
Proto-porphyrinogen IX
Proto-porphyrin IX
Protoheme (Heme)
Heme O
Heme A
Biliverdin-IX-δ
Biliverdin-IX-β
5-Oxo-δ-bilirubin
Peroxidase
Myoglobin
Cytochrome a
Cytochrome c
Catalase
Hemoglobin
Oxyhemoglobin
Biliverdin
(3Z)-Phytochromobilin
(3Z)-Phycocyanobilin
15,16-Dihydro-biliverdin
(3Z)-Phycoerythrobilin
Bilirubin
Bilirubin β-diglucuronide
D-Urobilinogen
D-Urobilin
I-Urobilinogen
I-Urobilin
L-Stercobilinogen
L-Stercobilin
CysG
CobA
(Anaerobic pathway)
(Aerobic pathway)
Sirohydrochlorin
Precorrin 2
Siroheme
Glutamate-1-semialdehyde
Glycine
L-Glutamyl-tRNA(Glu)
L-Glutamate
Ala, Asp and Glu metabolism
Ni-sirohydro-chlorin
Ni-sirohydro-chlorin a,c-diamide
15,17³-Seco-F 430-17³-acid
Coenzyme F 430
CbiK
CbiX
Co-sirohydrochlorin
CbiL
CobI
Precorrin 3A
CobG
Precorrin 3B
Co-factor 3
CbiH
CobJ
Co-precorrin 4
Precorrin 4
CbiF
CobM
Co-precorrin 5A
Precorrin 5
CbiG
Co-precorrin 5B
CobF
CbiD
Co-precorrin 6A
Precorrin 6A
CbiJ
CobK
Co-precorrin 6B
Precorrin 6B
CbiT
Co-precorrin 7
CobL
CbiE
Co-precorrin 8
Precorrin 8X
CbiC
CobH
Cobyrinate
Hydrogenobyrinate
CbiA
CobB
Hydrogenobyrinate a,c diamide
Cob(II)yrinate a,c diamide
CobNST
Cob(I)yrinate a,c diamide
CobA
BtuR
CobO
Adenosyl cobyrinate a,c diamide
CbiP
CobQ
Adenosyl cobyrinate hexaamide
(R)-1-Amino-propan-2-ol
CbiB
CobC
CobD
protein α
CbiZ
Adenosyl cobinamide
Cobinamide
CobU
CobP
Adenosyl cobinamide phosphate
L-Threonine phosphate
(R)-1-Amino-propan-2-yl phosphate
Glycine, threonine metabolism
L-Threonine
Cyanocob(III)alamin
CbiR
Adenosine-GDP-cobinamide
CobC
CobU
CobT
Dimethyl-benzimidazole
Bza
BzaD
BzaC
BzaAB
BzaF
5-Methoxy-benzimidazole
5-Hydroxy-benzimidazole
Aminoimidazole-ribotide
Purine metabolism
α-Ribazole
α-Ribazole 5'-P
5-Methoxy-6-methyl-benzimidazole
Riboflavin metabolism
CobS
CobV
Vitamin B12r
Aquacob(III)alamin
Vitamin B12s
Vitamin B12 coenzyme
Mg-protoporphyrin IX
Mg-protoporphyrin IX 13-monomethyl ester
(Anaerobic pathway)
(Aerobic pathway)
Zn-Bacterio-chlorophyll a
13¹-Hydroxy-Mg-protoporphyrin IX 13-monomethyl ester
13¹-Oxo-Mg-protoporphyrin IX 13-monomethyl ester
Divinylproto-chlorophyllide
Protochlorophyllide
BchJ
12-Ethyl-8-isobutyl-3-vinyl-bacteriochlorophyllide d
Bacterio-chlorophyllide e
12-Ethyl-8-propyl-3-vinyl-bacteriochlorophyllide d
8,12-Diethyl-3-vinyl-bacteriochlorophyllide d
Bacterio-chlorophyllide c
BchK
Bacterio-chlorophyll c
8-Ethyl-12-methyl-3-vinyl-bacteriochlorophyllide d
Bacterio-chlorophyllide d
Bacterio-chlorophyll d
3-Hydroxyethyl-chlorophyllide a
3-Hydroxyethyl-bacteriochlorophyllide a
Bacterio-chlorophyllide b
Geranylgeranyl-bacteriochlorophyllide b
Bacterio-chlorophyll b
Bacterio-pheophytins
3-Vinylbacterio-chlorophyllide a
Bacterio-chlorophyllide a
Geranylgeranyl-bacteriochlorophyllide a
Bacterio-chlorophyll a
Divinyl-chlorophyllide a
Chlorophyllide a
Pheophorbide a
Red chlorophyll catabolite
7-Hydroxy-chlorophyllide a
Chloro-phyllide b
Pyro-pheophorbide a
Primary fluorescent chlorophyll catabolite
7-Hydroxy-chlorophyll a
Divinyl-chlorophyll a
Chlorophyll a
Chlorophyll b
Pheophytin a
00860 1/20/22
(c) Kanehisa Laboratories

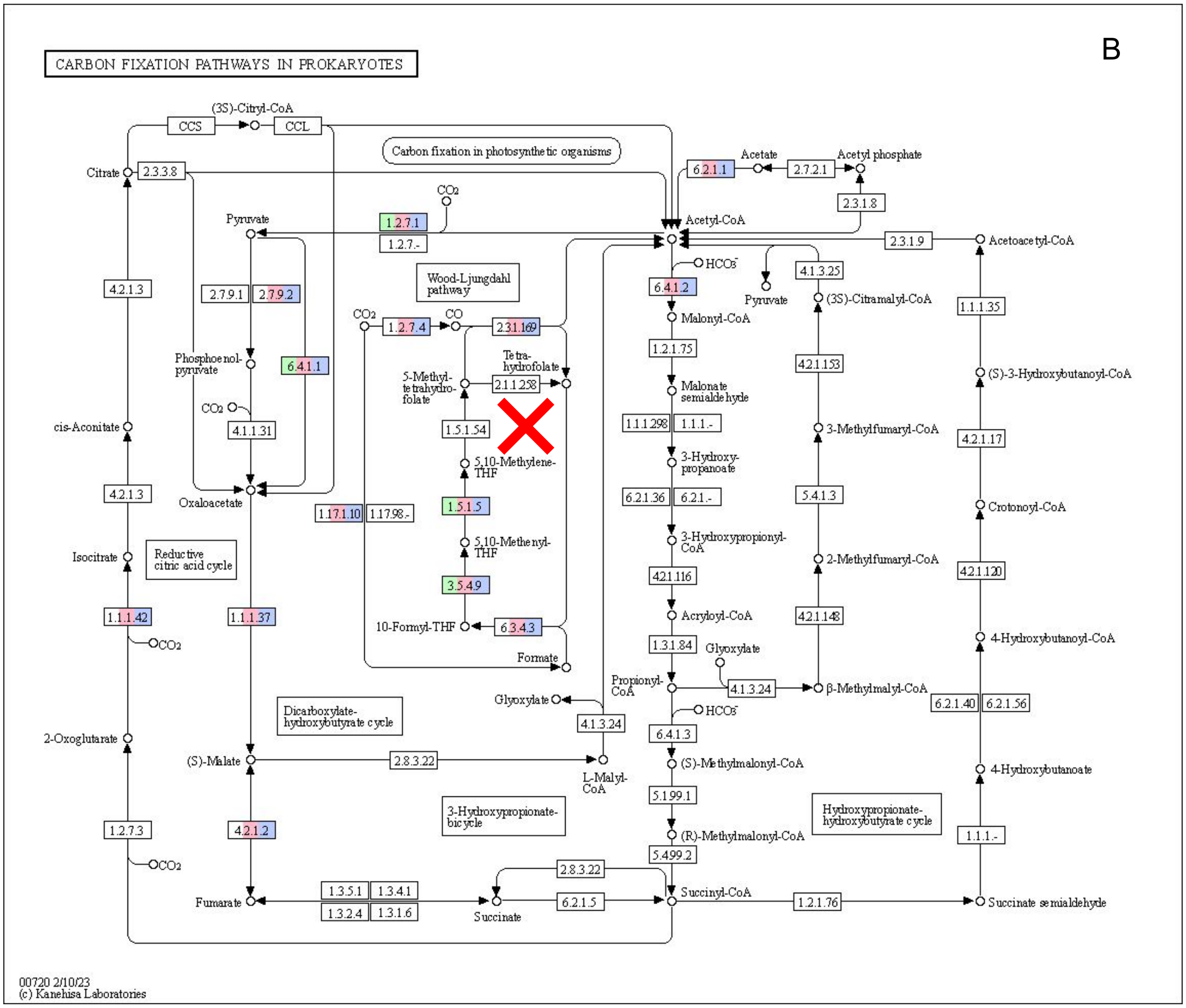

Figure S10. Corrinoid biosynthesis (A) and Wood-Ljungdahl pathway (B) in *Dehalococcides* MAGs. The presence of a gene in *Dehalococcides* MAGs recovered from F4-1, F4-2 and co-assembly is indicated by green, red, and blue. Key missing genes are marked nearby with a red "X". Key intermediates are marked with a purple rectangle. KEGG pathway[17] images are reproduced by permission of the Copyright holder Kanehisa Laboratories.

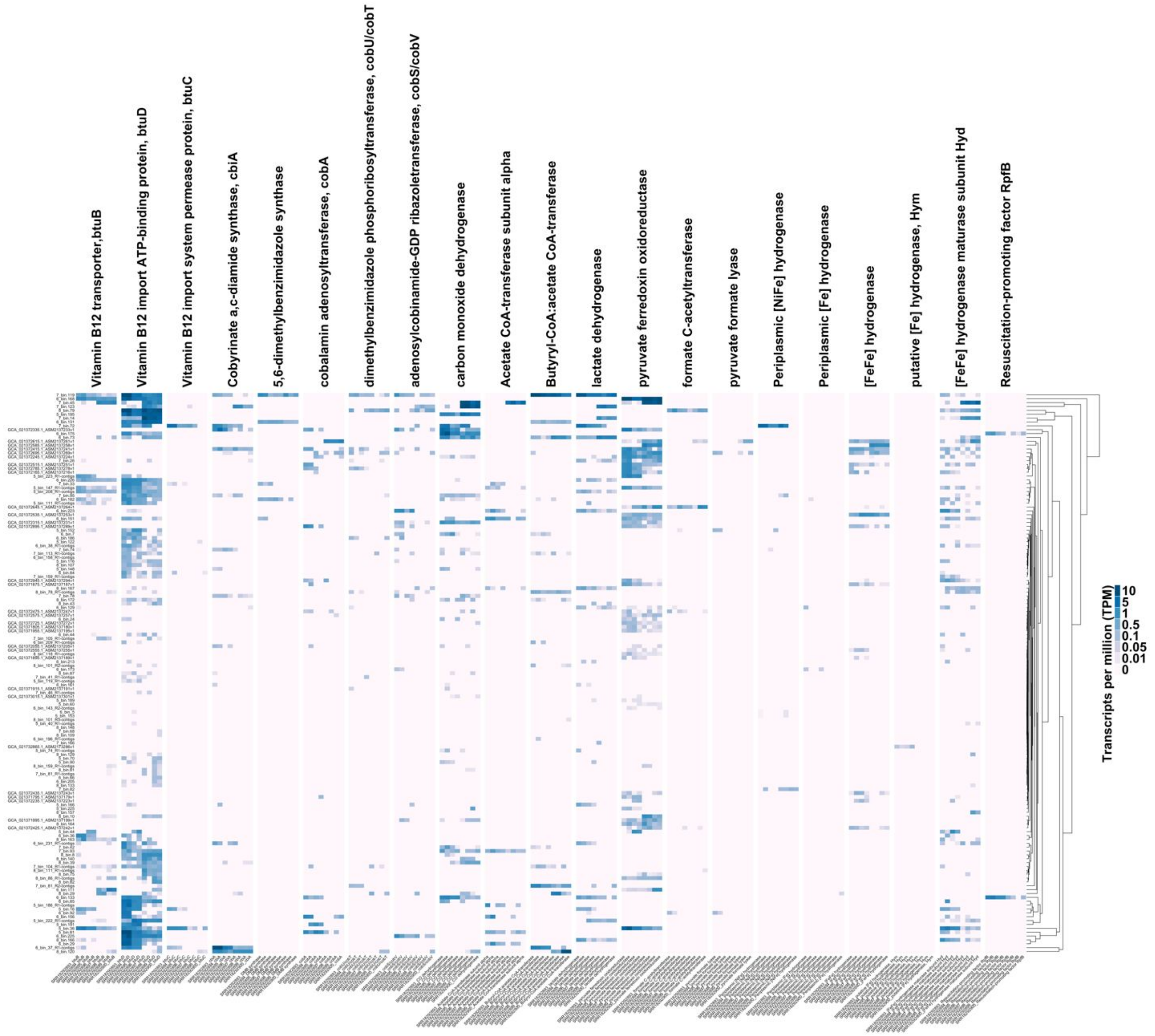

Figure S11. Each panel for the heatmap of transcripts per million showing the expression of *btuB*, *btuC*, *btuD* (btu associated with corrinoid transporting), *cbiA* (synthesize cobyrinate a,c-diamide), 5,6-dimethylbenzimidazole synthase (synthesize 5,6-dimethylbenzimidazole (DBM)), *cobA* (transform cobyrinate a,c-diamid/cobinamide), *cobU*/*cobT* (transform DBM) and *cobS/cobV* (synthesize corrinoid), carbon monoxide dehydrogenase (transform CO), Acetate CoA-transferase subunit alpha and Butyryl-CoA:acetate CoA-transferase (transform butyrate), lactate dehydrogenase (transform lactate), pyruvate ferredoxin oxidoreductase, formate C-acetyltransferase, pyruvate formate lyase (transform pyruvate), five hydrogenases and resuscitation-promoting factor *rpfB* from left to right. The MAGs were clustered based on the distribution of all genes. The data for generating this plot is provided in Table S7.

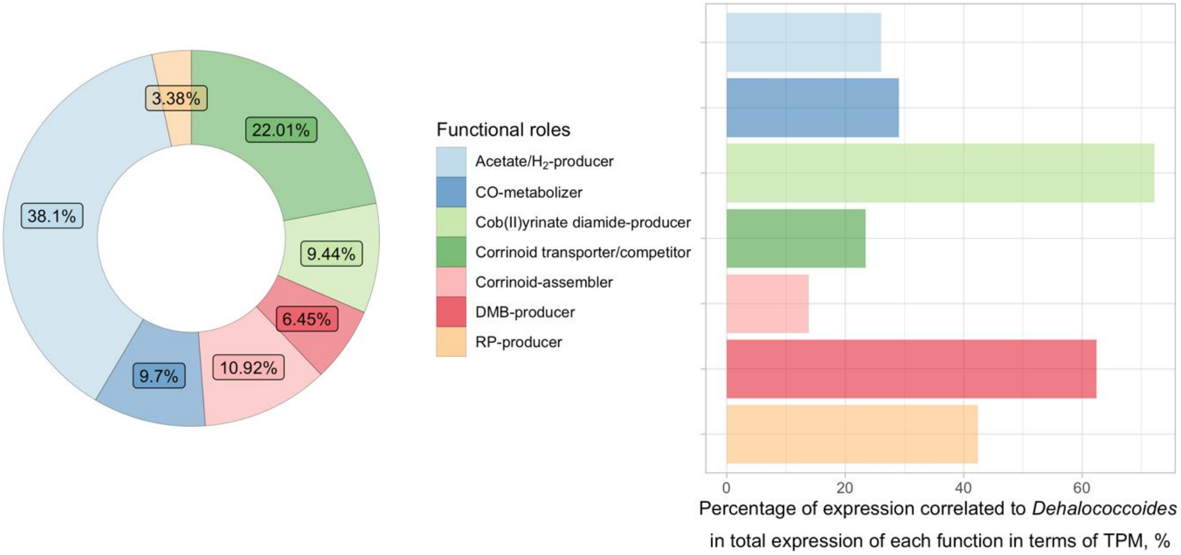

Figure S12. The total expression, as a percentage of TPM, of each role from MAGs related to *Dehalococcoides* (left), and the percent expression of TPM related to *Dehalococcoides* from all MAGs in each role (right).

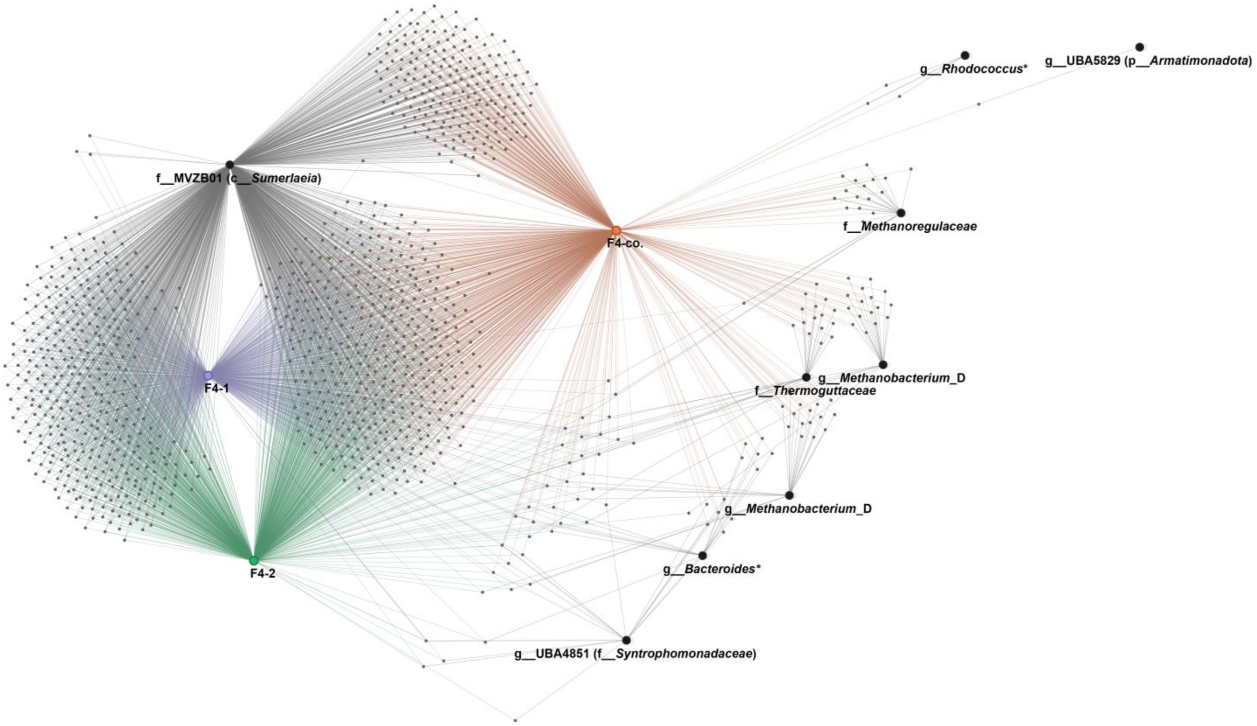

Figure S13. Expressed genes from MAGs without an assigned functional role that are correlated to expressed genes in the *Dehalococcoides* MAGs. The three *Dehalococcoides* MAGs (F4-1, F4-2, and F4 co-assembled (F4-co.) are colored in purple, green, and orange, respectively. The MAGs without assigned functional roles are in black with their assigned taxonomy. The smaller nodes represent the expressed genes within each MAG that are connected in this network analysis.

References

(1) Ewald Jessica, M.; Schnoor Jerald, L.; Mattes Timothy, E., Metagenomes, Metagenome-Assembled Genomes, and Metatranscriptomes from Polychlorinated Biphenyl-Contaminated Sediment Microcosms. *Microbiology Resource Announcements* **2022,** *11*, (7), e01126-21.

(2) Richards, P. M.; Mattes, T. E., Detection of an alkene monooxygenase in vinyl chloride-oxidizing bacteria with GeneFISH. *J Microbiol Methods* **2021,** *181*, 106147.

(3) Ewald, J. M.; Humes, S. V.; Martinez, A.; Schnoor, J. L.; Mattes, T. E., Growth of *Dehalococcoides* spp. and increased abundance of reductive dehalogenase genes in anaerobic PCB-contaminated sediment microcosms. *Environ Sci Pollut Res* **2020,** *27*, 8846-8858.

(4) Bustin, S. A.; Benes, V.; Garson, J. A.; Hellemans, J.; Huggett, J.; Kubista, M.; Mueller, R.; Nolan, T.; Pfaffl, M. W.; Shipley, G. L.; Vandesompele, J.; Wittwer, C. T., The MIQE Guidelines: Minimum Information for Publication of Quantitative Real-Time PCR Experiments. *Clinical Chemistry* **2009,** *55*, (4), 611-622.

(5) Mattes, T. E.; Ewald, J. M.; Liang, Y.; Martinez, A.; Awad, A.; Richards, P.; Hornbuckle, K. C.; Schnoor, J. L., PCB dechlorination hotspots and reductive dehalogenase genes in sediments from a contaminated wastewater lagoon. *Environ Sci Pollut Res* **2018,** *25*, (17), 16376-16388.

(6) Chaumeil, P.-A.; Mussig, A. J.; Hugenholtz, P.; Parks, D. H., GTDB-Tk: a toolkit to classify genomes with the Genome Taxonomy Database. *Bioinformatics* **2020,** *36*, (6), 1925-1927.

(7) Hyatt, D.; Chen, G.-L.; LoCascio, P. F.; Land, M. L.; Larimer, F. W.; Hauser, L. J., Prodigal: prokaryotic gene recognition and translation initiation site identification. *BMC Bioinformatics* **2010,** *11*, (1), 119.

(8) Eddy, S. R., Accelerated Profile HMM Searches. *PLOS Computational Biology* **2011,** *7*, (10), e1002195.

(9) Matsen, F. A.; Kodner, R. B.; Armbrust, E. V., pplacer: linear time maximum-likelihood and Bayesian phylogenetic placement of sequences onto a fixed reference tree. *BMC Bioinformatics* **2010,** *11*, (1), 538.

(10) Jain, C.; Rodriguez-R, L. M.; Phillippy, A. M.; Konstantinidis, K. T.; Aluru, S., High throughput ANI analysis of 90K prokaryotic genomes reveals clear species boundaries. *Nature Communications* **2018,** *9*, (1), 5114.

(11) Price, M. N.; Dehal, P. S.; Arkin, A. P., FastTree 2 – Approximately Maximum-Likelihood Trees for Large Alignments. *PLOS ONE* **2010,** *5*, (3), e9490.

(12) Sievers, F.; Wilm, A.; Dineen, D.; Gibson, T. J.; Karplus, K.; Li, W.; Lopez, R.; McWilliam, H.; Remmert, M.; Söding, J.; Thompson, J. D.; Higgins, D. G., Fast, scalable generation of high-quality protein multiple sequence alignments using Clustal Omega. *Molecular Systems Biology* **2011,** *7*, (1), 539.

(13) Minh, B. Q.; Schmidt, H. A.; Chernomor, O.; Schrempf, D.; Woodhams, M. D.; von Haeseler, A.; Lanfear, R., IQ-TREE 2: New Models and Efficient Methods for Phylogenetic Inference in the Genomic Era. *Molecular Biology and Evolution* **2020,** *37*, (5), 1530-1534.

(14) Letunic, I.; Bork, P., Interactive Tree Of Life (iTOL) v5: an online tool for phylogenetic tree display and annotation. *Nucleic Acids Research* **2021,** *49*, (W1), W293-W296.

(15) Seemann, T., Prokka: rapid prokaryotic genome annotation. *Bioinformatics* **2014,** *30*, (14), 2068-2069.
(16) Aramaki, T.; Blanc-Mathieu, R.; Endo, H.; Ohkubo, K.; Kanehisa, M.; Goto, S.; Ogata, H., KofamKOALA: KEGG Ortholog assignment based on profile HMM and adaptive score threshold. *Bioinformatics* **2020,** *36*, (7), 2251-2252.
(17) Kanehisa, M.; Furumichi, M.; Sato, Y.; Kawashima, M.; Ishiguro-Watanabe, M., KEGG for taxonomy-based analysis of pathways and genomes. *Nucleic Acids Research* **2023,** *51*, (D1), D587-D592.
(18) Kanehisa, M.; Sato, Y.; Kawashima, M., KEGG mapping tools for uncovering hidden features in biological data. *Protein Science* **2022,** *31*, (1), 47-53.
(19) Delmont, T. O.; Eren, A. M., Linking pangenomes and metagenomes: the Prochlorococcus metapangenome. *PeerJ* **2018,** *6*, e4320.
(20) Pritchard, L.; Glover, R. H.; Humphris, S.; Elphinstone, J. G.; Toth, I. K., Genomics and taxonomy in diagnostics for food security: soft-rotting enterobacterial plant pathogens. *Analytical Methods* **2016,** *8*, (1), 12-24.
(21) Camacho, C.; Coulouris, G.; Avagyan, V.; Ma, N.; Papadopoulos, J.; Bealer, K.; Madden, T. L., BLAST+: architecture and applications. *BMC Bioinformatics* **2009,** *10*, (1), 421.
(22) Mistry, J.; Chuguransky, S.; Williams, L.; Qureshi, M.; Salazar, Gustavo A.; Sonnhammer, E. L. L.; Tosatto, S. C. E.; Paladin, L.; Raj, S.; Richardson, L. J.; Finn, R. D.; Bateman, A., Pfam: The protein families database in 2021. *Nucleic Acids Research* **2021,** *49*, (D1), D412-D419.
(23) Molenda, O.; Puentes Jácome, L. A.; Cao, X.; Nesbø, C. L.; Tang, S.; Morson, N.; Patron, J.; Lomheim, L.; Wishart, D. S.; Edwards, E. A., Insights into origins and function of the unexplored majority of the reductive dehalogenase gene family as a result of genome assembly and ortholog group classification. *Environmental Science: Processes & Impacts* **2020,** *22*, (3), 663-678.
(24) Mirdita, M.; Schütze, K.; Moriwaki, Y.; Heo, L.; Ovchinnikov, S.; Steinegger, M., ColabFold: making protein folding accessible to all. *Nature Methods* **2022,** *19*, (6), 679-682.
(25) Pettersen, E. F.; Goddard, T. D.; Huang, C. C.; Meng, E. C.; Couch, G. S.; Croll, T. I.; Morris, J. H.; Ferrin, T. E., UCSF ChimeraX: Structure visualization for researchers, educators, and developers. *Protein Science* **2021,** *30*, (1), 70-82.
(26) Yoshida, N.; Takahashi, N.; Hiraishi, A., Phylogenetic characterization of a polychlorinated-dioxin-dechlorinating microbial community by use of microcosm studies. *Applied and environmental microbiology* **2005,** *71*, (8), 4325-4334.
(27) Johnson, L. A.; Hug, L. A., Cloacimonadota metabolisms include adaptations in engineered environments that are reflected in the evolutionary history of the phylum. *Environmental Microbiology Reports* **2022,** *14*, (4), 520-529.
(28) Wood, D. E.; Lu, J.; Langmead, B., Improved metagenomic analysis with Kraken 2. *Genome Biol* **2019,** *20*, (1), 1-13.
(29) Wang, S.; Chen, C.; Zhao, S.; He, J., Microbial synergistic interactions for reductive dechlorination of polychlorinated biphenyls. *Science of The Total Environment* **2019,** *666*, 368-376.

(30) Ding, C.; Rogers, M. J.; Yang, K. L.; He, J., Loss of the *ssrA* genome island led to partial debromination in the PBDE respiring *Dehalococcoides mccartyi* strain GY50. *Environ Microbiol* **2017,** *19*, (7), 2906-2915.
(31) Wang, S.; Chng, K. R.; Chen, C.; Bedard, D. L.; He, J., Genomic characterization of *Dehalococcoides mccartyi* strain JNA that reductively cechlorinates tetrachloroethene and polychlorinated biphenyls. *Environ Sci Technol* **2015,** *49*, (24), 14319-14325.
(32) Parks, D. H.; Rinke, C.; Chuvochina, M.; Chaumeil, P.-A.; Woodcroft, B. J.; Evans, P. N.; Hugenholtz, P.; Tyson, G. W., Recovery of nearly 8,000 metagenome-assembled genomes substantially expands the tree of life. *Nature Microbiology* **2017,** *2*, (11), 1533-1542.
(33) Zhao, S.; Rogers, M. J.; Cao, L.; Ding, C.; He, J., Identification of reductive dehalogenases that mediate complete debromination of penta-and tetra-brominated diphenyl ethers in *Dehalococcoides* spp. *Appl Environ Microbiol* **2021,** *87*, (17), e00602-21.
(34) Wang, S.; Chng, K. R.; Wilm, A.; Zhao, S.; Yang, K.-L.; Nagarajan, N.; He, J., Genomic characterization of three unique *Dehalococcoides* that respire on persistent polychlorinated biphenyls. *Proc Nat Acad Sci USA* **2014,** *111*, (33), 12103-12108.
(35) Ewald, J. M.; Schnoor, J. L.; Mattes, T. E., Combined read- and assembly-based metagenomics to reconstruct a Dehalococcoides mccartyi genome from PCB-contaminated sediments and evaluate functional differences among organohalide-respiring consortia in the presence of different halogenated contaminants. *FEMS Microbiology Ecology* **2022,** *98*, (7), fiac067.
(36) Müller, N.; Worm, P.; Schink, B.; Stams, A. J. M.; Plugge, C. M., Syntrophic butyrate and propionate oxidation processes: from genomes to reaction mechanisms. *Environmental Microbiology Reports* **2010,** *2*, (4), 489-499.
(37) Walker, C. B.; He, Z.; Yang, Z. K.; Ringbauer, J. A., Jr.; He, Q.; Zhou, J.; Voordouw, G.; Wall, J. D.; Arkin, A. P.; Hazen, T. C.; Stolyar, S.; Stahl, D. A., The electron transfer system of syntrophically grown Desulfovibrio vulgaris. *J Bacteriol* **2009,** *191*, (18), 5793-801.
(38) Löffler, F. E.; Yan, J.; Ritalahti, K. M.; Adrian, L.; Edwards, E. A.; Konstantinidis, K. T.; Müller, J. A.; Fullerton, H.; Zinder, S. H.; Spormann, A. M., *Dehalococcoides mccartyi* gen. nov., sp. nov., obligately organohalide-respiring anaerobic bacteria relevant to halogen cycling and bioremediation, belong to a novel bacterial class, *Dehalococcoidia* classis nov., order *Dehalococcoidales* ord. nov. and family *Dehalococcoidaceae* fam. nov., within the phylum *Chloroflexi*. *Int J Syst Evol Microbiol* **2013,** *63*, (2), 625-635.
(39) Yan, J.; Şimşir, B.; Farmer, A. T.; Bi, M.; Yang, Y.; Campagna, S. R.; Löffler, F. E., The corrinoid cofactor of reductive dehalogenases affects dechlorination rates and extents in organohalide-respiring *Dehalococcoides mccartyi*. *ISME J* **2016,** *10*, (5), 1092-1101.
(40) Men, Y.; Yu, K.; Bælum, J.; Gao, Y.; Tremblay, J.; Prestat, E.; Stenuit, B.; Tringe, S. G.; Jansson, J.; Zhang, T., Metagenomic and metatranscriptomic analyses reveal the structure and dynamics of a dechlorinating community containing *Dehalococcoides mccartyi* and corrinoid-providing microorganisms under cobalamin-limited conditions. *Appl Environ Microbiol* **2017,** *83*, (8).
(41) Cadieux, N.; Bradbeer, C.; Reeger-Schneider, E.; Köster, W.; Mohanty Arun, K.; Wiener Michael, C.; Kadner Robert, J., Identification of the Periplasmic Cobalamin-Binding Protein BtuF of Escherichia coli. *Journal of Bacteriology* **2002,** *184*, (3), 706-717.

(42) Zhuang, W.-Q.; Yi, S.; Bill, M.; Brisson, V. L.; Feng, X.; Men, Y.; Conrad, M. E.; Tang, Y. J.; Alvarez-Cohen, L., Incomplete Wood–Ljungdahl pathway facilitates one-carbon metabolism in organohalide-respiring Dehalococcoides mccartyi. *Proceedings of the National Academy of Sciences* **2014,** *111*, (17), 6419-6424.

(43) Su, X.; Xie, M.; Han, Z.; Xiao, Y.; Wang, R.; Shen, C.; Hashmi Muhammad, Z.; Sun, F., Resuscitation-Promoting Factor Accelerates Enrichment of Highly Active Tetrachloroethene/Polychlorinated Biphenyl-Dechlorinating Cultures. *Applied and Environmental Microbiology* **2023,** *89*, (1), e01951-22.
